# Calixarenes and Their Biomimetic Applications

**DOI:** 10.1155/S1565363304000159

**Published:** 2004

**Authors:** Y. K. Agrawal, Harshit Bhatt

**Affiliations:** Institute of Pharmacy and Sciences Nirma University of Science & Technology Sarkhej Gandhinagar Highway Ahmedabad 382481 India

## Abstract

The synthetic models for the structures, spectroscopic properties and catalytic activities of metalloprotein active sites have been reviewed. Calixarenes were used as new biomimetic catalysts because of their advantage of providing preorganiiation of the catalytic group, which can bind the substrate dynamically that results in fast turnover and fast release of the products. Functional and structural models based on calixarenes are presented and in addition importance of molecular recognition and non-covalent interactions e.g. hydrogen bonding and their role in biological systems are discussed with the help of synthetic systems.


### Calixarenes and Their Biomimetic Applications

Y.K. Agrawal and Harshit Bhatt
*Institute of Pharmacy and Sciences, Nirma University of Science & Technology*
*Sarkhej Gandhinagar Highway, Ahmedabad 382481, India*
*E-mail: drykagrawal@yahoo.com*

### CONTENTS

Abstract
1. Introduction
2. Biomimetism based on function
   2.1 Zn(II) complexes: Hydrolytic metalloenzymes
   2.2 Cu(II) complexes: Hydrolytic metalloenzymes
   2.3 Acyltransferase enzyme mimic
   2.4 Ribonuclease enzyme mimic
   2.5 ATPase enzyme mimic
   2.6 Aldolase enzyme mimic
   2.7 Heme mimics
   2.8 Carbonic anhydrase enzyme mimic
3. Biomimetism based on structure
   3.1 Importance of Cu(I) and Cu(II) in biological systems
   3.2 Calixarene based Cu(I) and Cu(II) complexes as structural models
   3.3 Importance of Zn(II) in biological systems
   3.4 Calixarene based Zn(II) complexes as structural models
4. Biomimetism based on molecular recognition and importance of hydrogen bonding
5. Concluding remarks

Acknowledgements
Reference

### ABSTRACT

The synthetic models for the structures, spectroscopic properties and catalytic activities of metalloprotein active sites have been reviewed. Calixarenes were used as new biomimetic catalysts because of their advantage of providing preorganization of the catalytic group, which can bind the substrate dynamically that results in fast turnover and fast release of the products. Functional and structural models based on calixarenes are presented and in addition importance of molecular recognition and non-covalent interactions e.g.

hydrogen bonding and their role in biological systems are discussed with the help of synthetic systems.

### 1. INTRODUCTION

Supramolecular chemistry /1,2/ is dominated by forces resulting from intermolecular non-bonded, non-covalent electrostatic forces and dispersion forces, whose development requires the use of all resources of molecular chemistry combined with a designed manipulation of noncovalent interactions so as to form supramolecular entities. Many efforts have been made to design the appropriate model or mimic for biological processes. Macrocyclic receptors meet some of the requirements for designing artificial receptors like:

- They possess cavities and clefts of appropriate sizes and shapes
- Reactive binding sites
- Branches and bridges
- Connections (polycyclic) that allow the construction of a desired architecture.

One such class of compounds 'Calixarenes' was investigated which has historical roots in the 19th century and it has been suggested as a potential enzyme mimic /3/. The widespread research on molecular recognition of guest by synthetic hosts has stimulated bio(in)organic chemists to design catalysts that mimic the active sites of enzymes. This aspect of supramolecular chemistry involves the synthesis of enzyme models or artificial enzymes that have the same catalytic function but are structurally less complex and more stable than enzymes. Such enzyme models can provide information on mechanistic aspects of enzyme action and may find future application as catalyst in industrial synthesis. Foremost among the compounds investigated for this purpose are cyclodextrins whose torus shape endows them with the ability to form host-guest complexes and in certain cases to act as powerful catalysts /4/. However, these compounds have a disadvantage of being available in only a limited array of sizes. Therefore, calixarenes are of great interest, since they possess an architecture similar to that of the cyclodextrins, but are amenable to greater variation and control. These are particularly attractive components for attempting to construct *in vitro* systems that mimic *in vivo* catalytic activity of the enzymes. They are versatile molecular scaffolds for the design of host molecules because the functional groups are directionally preorganized. They can also create a binding site in which functional groups can dynamically adjust to the guest by low energy conformational changes of the calixarene skeleton. Although many calix[4]arene-based molecular receptors have been reported /5,6/,there have been few developments with regard to calixarene enzyme-models /7-12/.

A synthetic model system possesses the following features shown by enzymes:

- Fast reaction rate under mild conditions
- High degree of structural and chiral recognition of their substrates
- High turnover rate for a number of substrates without being destroyed.
- May be inhibited by the compounds that bind to the enzyme but do not react themselves.

In addition to these features, enzyme mimics should be less complex, molecular weight less than natural enzymes, stable even at high temperatures and soluble in most of the solvents. Biological reactions found in living nature can be mimicked in purely chemical conditions, particularly with small and simple substrates. The discovery of the biomimetic reactions may not lead to very efficient system, but then on their development, lead to more perfect states from the knowledge of their mechanisms and using chemical means as well as principles of biological systems with complex organization.

The following two main approaches may be adopted from several strategies to understand high-complex biological processes, which nature has spent millions of years to evolve in their present forms:

1. Functional model- A system (intermolecular in general) that carries out the reaction concerned without the binding characteristics of the enzyme and may perform functional aspects of the natural enzymes.
2. Structural model- A system that shows efficient binding characteristics of the substrate or simplified analogue, or more properly an analogue of the transition state of the biological reaction.

### 2. BIOMIMETISM BASED ON FUNCTION

The functional properties of supramolecular systems are highly dependent on two major features: (i) reactivity and (ii) catalysis. Molecular receptors bearing appropriate reactive groups in addition to binding sites may complex a substrate (with given stability, selectivity, and kinetic features), react with it (with given rate, selectivity, and turnover), and release the products, thus regenerating the reagent for a new cycle. The design of efficient and selective supramolecular reagents and catalysts may give mechanistic insight into the elementary steps of catalysis, provide new types of chemical reagents, and effect reactions that reveal factors contributing to enzymatic catalysis. A large number of reagents based on functionalized cyclodextrins, macrocyclic polyethers and calixarenes have been investigated.

Ester cleavage processes have been most frequently investigated in enzyme model studies. In nature, chemical transformations of phosphate esters by metalloenzymes are generally facilitated by the co-operative action of two metal ions, e.g. in P1 nuclease, DNA polymerase I, phospholipase C, and alkaline phosphatase. Zn(II), Mg(II), Mn(II) or Fe(III) are usually found in such enzymes with 3-5 $A^0$ distance in the active site. The phosphate group is activated due to the Lewis acid function of these metal ions, which generate reactive nucleophile and stabilize both the pentacoordinate phosphorous transition state and the leaving group. Several artificial active site models have been reported in which two metal centers such as Zn(II) /7-13/, Cu(II) /14/, Co(III) /15/ and lanthanides(III) /16/ are held apart by two ligands that are linked to a molecular scaffold. An appropriate molecular scaffold is required for spatial organization to design an efficient enzyme mimic. Attempts have been made in the past decades to catalyze biochemical reactions with synthetic compounds in which appropriate catalytic groups are oriented in such a way that the transition state of the reaction can be stabilized. However, low activity is generally observed compared to catalytic activity of enzymes. Enzymes exhibit a certain degree of flexibility and can bind the substrate and transition state by an induced fit mechanism, whereas biomimetic compounds are often too rigid or too flexible. Designed active site models for dinuclear metallophosphodiesterases are often dinuclear transition metal complexes in which a molecular spacer oriented two catalytic metal ions. The choice of spacer affects the catalytic properties. A very flexible spacer may result in a lack of catalytic cooperativity between the metal centers. In contrast, very rigid molecular scaffold might not be able to match the geometrical changes of the substrate during the dynamic catalytic process. Thus, to be an efficient enzyme mimic, such models require molecular scaffold, that allow both a proper organization of multiple catalytic group and a certain degree of flexibility. Calixarenes might be suitable building blocks since multiple catalytic groups can be preorganized dynamically, similar to the amino acid residues and cofactors in the polypeptide backbone of enzymes. Nonenzymatic phosphate ester cleavage is effectively facilitated by the Co(III) /15,17/ and lanthanides (III) /16,17a,18/ ions. However, for application as artificial nucleases, the use of Co(III) is not attractive since it forms substitutional inert complexes with the products, which hamper turnover catalysis at neutral pH. The disadvantage of lanthanide(III) ions is their toxicity and the laborious formation of sufficiently stable complexes. Free lanthanide(III) ions form readily lanthanide(III) hydroxide precipitates, and both forms are active in phosphate ester cleavage, which makes kinetic control complicated. Although occasionally substantial rate accelerations for phosphate ester hydrolysis have been observed, low substrate binding and lack of turnover are general problems encountered with simple model systems /9/.

Generally; *p*-nitrophenyl esters have been used as substrates, because reaction rate can be easily determined by the measurement of the increase in absorbance at $\lambda$ = 400 nm due to the release of *p*-nitro phenolate on hydrolysis. Mainly, two model substrates were selected to study hydrolytic reactions using functionalized calixarenes as catalysts.

### (a) 2- Hydroxy propyl p-nitrophenyl phosphate(HPNP)

The substrate 2- hydroxypropyl *p*-nitrophenyl phosphate(HPNP, Fig. 1) is an RNA model substrate that reacts via intramolecular trans-esterification by nucleophilic attack of the β - hydroxy group.

### (b) Ethyl-p-nitrophenyl phosphate(EPNP)

Ethyl-*p*-nitrophenyl phosphate (EPNP, Fig. 2) can be considered as a DNA model substrate that reacts via intermolecular nucleophilic attack of a hydroxide ion.

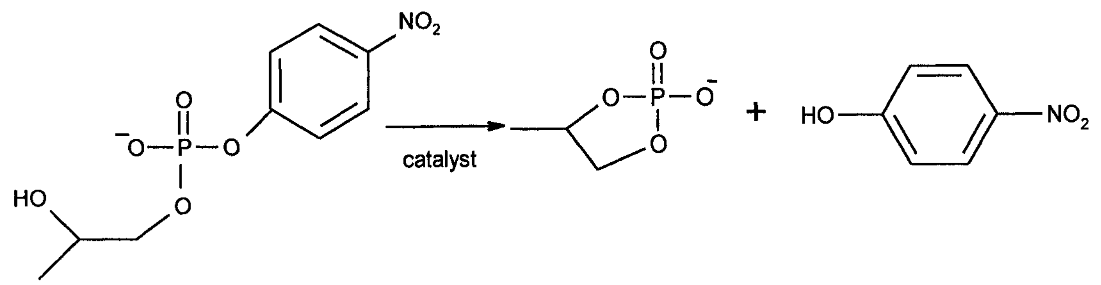

**Fig. 1:** RNA model – HPNP

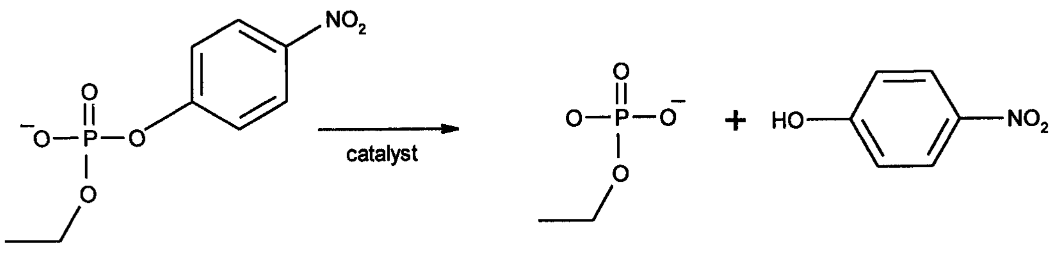

**Fig. 2:** DNA model – EPNP

### 2.1. Zn(II) complexes: Hydrolytic metalloenzymes

Upper rim functionalized calixarenes have been reported for the complexation of zinc and copper ions. Mono-, di- and trinuclear Zn(II)-calixarene complexes have been discussed /7,8,12,19/ as enzyme models for hydrolytic reactions and compared with pyridine-Zn reference complex (Fig. 4).

The mononuclear calix[4]arene complex (Fig. 3) is a factor 6 more active than reference complex (Fig. 4), which lacks the calix[4]arene backbone, indicating that the hydrophobic effect plays a role in the catalytic process. The calix[4]arene can assist in the catalysis via its hydrophobic aromatic surface that can lower the $pK_a$ of a nearby metal bound water molecule and can interact with the substrate. Both the binding of the substrate and the conversion of the substrate were found to be far more efficient in dinuclear complex (Fig. 5) compared to mononuclear complex (Fig. 3). The catalytic activity of mononuclear complex was found to be a factor of 50 lower, compare to dinuclear complex. The presence of 0.48 mM induces a 23000 fold, rate enhancement in the catalytic cyclization of the RNA-model substrate 2-hydroxy propyl *p*-nitrophenyl

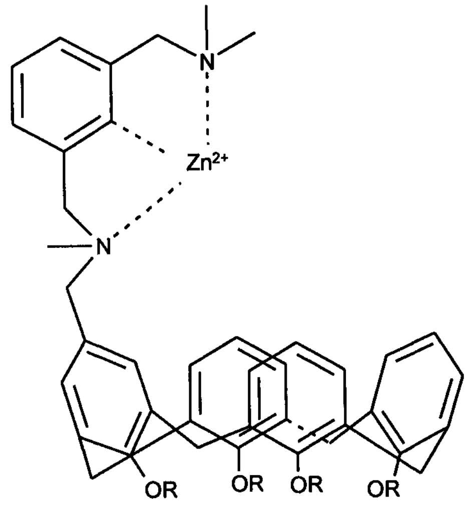

**Fig. 3:** Calix[4]arene based mono-nuclear Zn(II) models

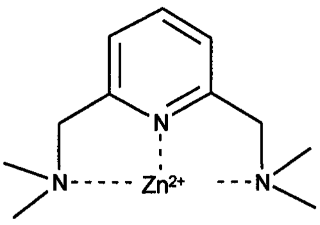

**Fig. 4:** Reference pyridine complex

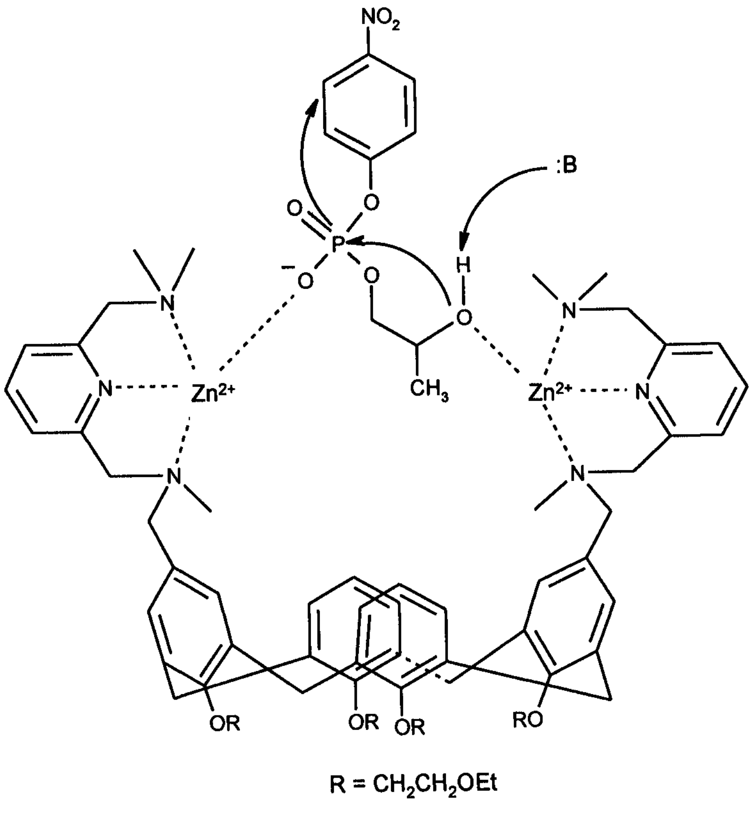

**Fig. 5:** Proposed mechanism for HPNP cleavage by calix[4]arene based dinuclear Zn(II) models phosphate (HPNP, pH 7.0, $25^0$ C), which is the largest catalytic rate acceleration reported for nuclease mimics. Comparison with mono functionalized calix[4]arene (Fig. 3) and reference pyridine zinc complex (Fig. 4) showed that the high catalytic activity can be attributed to a favourable contribution of the calixarene moiety in substrate binding and catalytic synergic action of the two Zn(II) centers.

Dinuclear Zn(II) Model (Fig. 5) was proposed to bind HPNP by two point coordination. One Zn(II) center activating the phosphoryl group and the other activating β-hydroxyl group, subsequently followed by general base promoted cyclization. The catalytic activity of dinuclear complex (Fig. 5) was compared with modified dinuclear complex (Fig. 6), which was rigidified by modification of the calix[4]arene lower rim with a crown

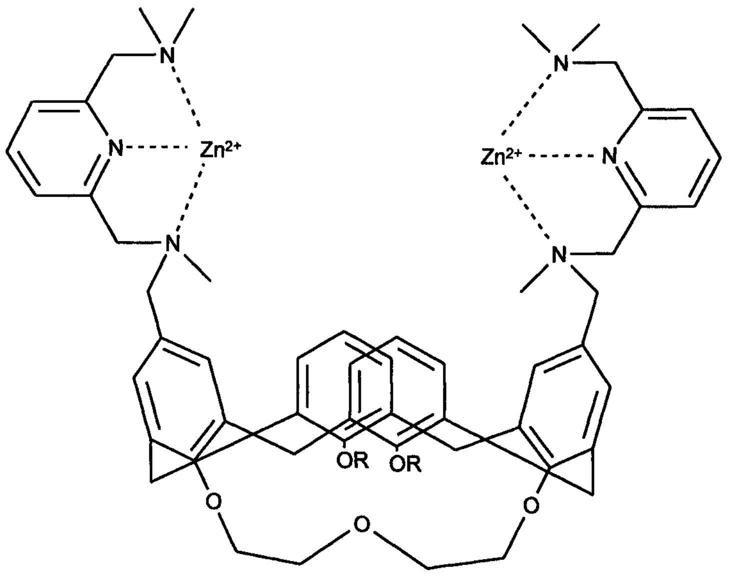

**Fig. 6:** Calix[4]arene based dinuclear Zn(II) models bridge and it was found to be less efficient by the factor of 8, than flexible dinuclear complex in both HPNP binding and conversion which showed the importance of flexibility in the cooperative action of Zn(II) during catalysis.

Phosphodiesterase enzymes such as P1 nuclease use three divalent metal ions [e.g. Zn(II)] in the active site to catalyze the hydrolytic cleavage of phosphate diester bonds in nucleotides like DNA and RNA. Synthetic catalysts that cleave RNA at specific sites are of interest for future applications in gene technology.

The dinuclear complex was enlarged with an additional Zn(II) center to trinuclear Zn(II) complex (Fig. 7) mimicking the active site of trinuclear metallohydrolases /8/. The trinuclear Zn(II) model was reported as an efficient catalyst for the cleavage of RNA dinucleotides due to the cooperative effect of the Zn(II) centers with high rate enhancement and significant nucleobase specificity. The catalytic activity of this trinuclear complex was measured for a series of RNA-dinucleotides, which exhibited a very high catalytic activity and rate accelerations over the uncatalyzed reactions by the order of $10^4$- $10^5$. Large differences in rate for different nucleobases in the dinucleotides were also observed. Thus, the three Zn(II) ions have been found to catalyze the hydrolytic cleavage of phosphate diester bonds in phosphatidicholine and in nucleotides such as RNA and DNA, respectively. However, the function of a third metal ion in close proximity to a dinuclear metal cluster in enzyme is not yet fully understood, and only one example of a biomimetic study has been reported /13a/. Three Zn(II) centers are co-operatively involved in the catalysis. Double Lewis acid activation occurs by two neighboring Zn(II) centers and the third Zn(II) center can possibly facilitate deprotonation of the β-hydroxy group of HPNP, either by Lewis acid activation similar to catalysis by dinuclear complex or via a bound hydroxide ion. These models appeared to be more efficient catalysts, and the third Zn(II) center affects 40% higher rate enhancement in HPNP cleavage. The presence of the third Zn(II) center in close proximity to the dinuclear Zn(II) cluster results in a decreased substrate affinity and an increased catalytic

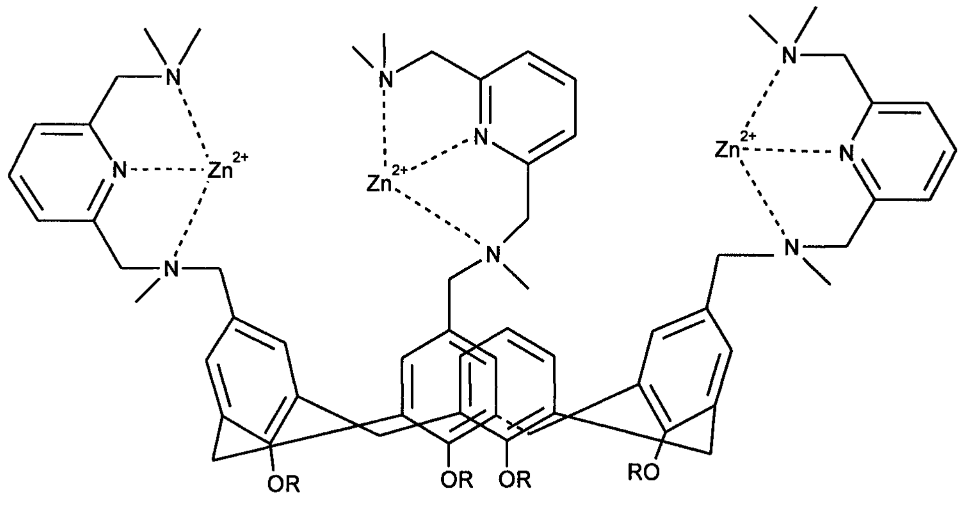

**Fig. 7:** Trinuclear Zn(II)-calix[4]arene complex as model for hydrolytic metalloenzymes rate. This implies that trinuclear complex binds the pentacoordinate phosphorous transition state better than dinuclear complex. The heterodinuclear complex containing two Zn(II) and one Cu(II) was found to be even more active. This might be due to synergy of the favourable properties of Zn(II) in the binding and Cu(II) in the conversion of the phosphate diester substrate.

### 2.2. Cu(II) complexes: Hydrolytic metalloenzymes

In the phosphoesterase metalloenzymes several amino acid residues are also involved in the catalysis. In the hydrolysis of phosphate monoesters, by alkaline phosphatase, two Zn(II) ions and one Mg(II) ion are assisted by a serine, a histidine and an arginine side chain. In RNase A, an ammonium group of a lysine residue activates a phosphoryl group, while two histidines co-operate in deprotonation of the nucleophile and the protonation of the leaving group /20/. The catalytic role of the amino acid residues in RNase A has been mimicked with general acid-base systems, like bisimidazoles and aqueous solutions of imidazoles or diamines /21/. Recently, poly(ethylene glycol)-supported Cu(II)- triazacyclononane was reported as an efficient, recoverable and recyclable catalyst for the cleavage of the phosphodiester /22/. Cis-diaqua Cu(II) complexes, either mononuclear /23-26/ or dinuclear /27,14/ have also been reported as active artificial catalysts for the cleavage of phosphate diesters. Chin has shown that the unique catalyst neocuproine –Cu(II) and a 1,8- naphthalene substituted dinuclear[9]ane-$N_3$-Cu(II) complex cleave RNA rapidly under mild conditions /24-26/. An enzyme mimic, the Cu(II) complex of a cyclodextrin dimer linked by a bipyridyl unit was reported by Breslow *et al.* which catalyzes the hydrolysis of an unactivated doubly-bound benzyl ester /28/.

The synthesis, characterization and catalytic activity of the dinuclear calix[4]arene-Cu(II) complex (Fig. 8) and the mono nuclear reference complex (Fig. 9) were described /9/ and shown as mimic for dinuclear

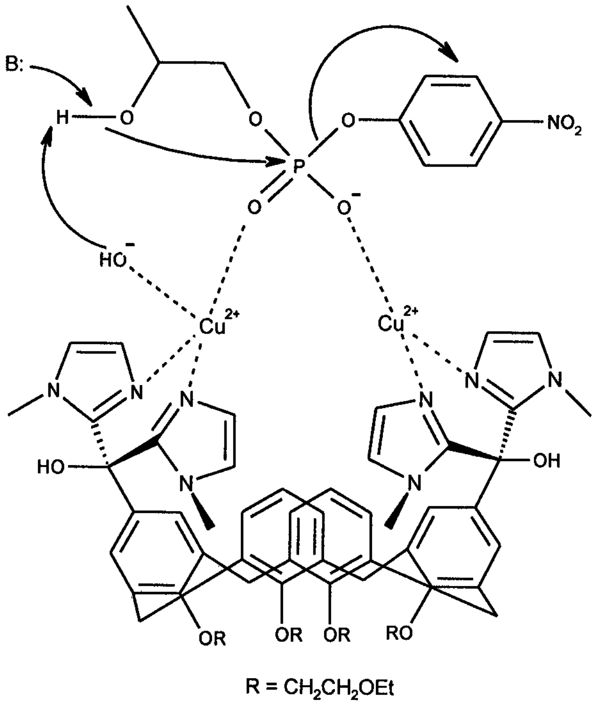

**Fig. 8:** Proposed mechanism for HPNP cleavage by calix[4]arene-Cu(II) complex

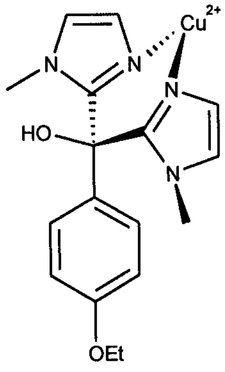

**Fig. 9:** Mononuclear reference complex metallophosphodiesterases. This novel calix[4]arene dinuclear-Cu(II) complex binds and cleaves the phosphate diesters HPNP ($1 \times 10^4$ rate enhancement) and EPNP ($2.7 \times 10^4$ rate enhancement) far more efficiently than the mononuclear reference complex. This is due to the efficient synergic action of the two Cu(II) centers, which are well-preorganized on the flexible calix[4]arene scaffold. Another interesting similiarity of this calix[4]arene based artificial metalloenzymes is the remarkably low $pK_a$ of the metal(II) bound water molecules, which makes the artificial enzymes active under the slightly acidic to neutral conditions. The low $pK_a$ is the result of local hydrophobic environment in the natural enzymes, created by the aromatic surface of the calix[4]arene moiety /7/. Bifunctional phosphodiesterase models that possess two

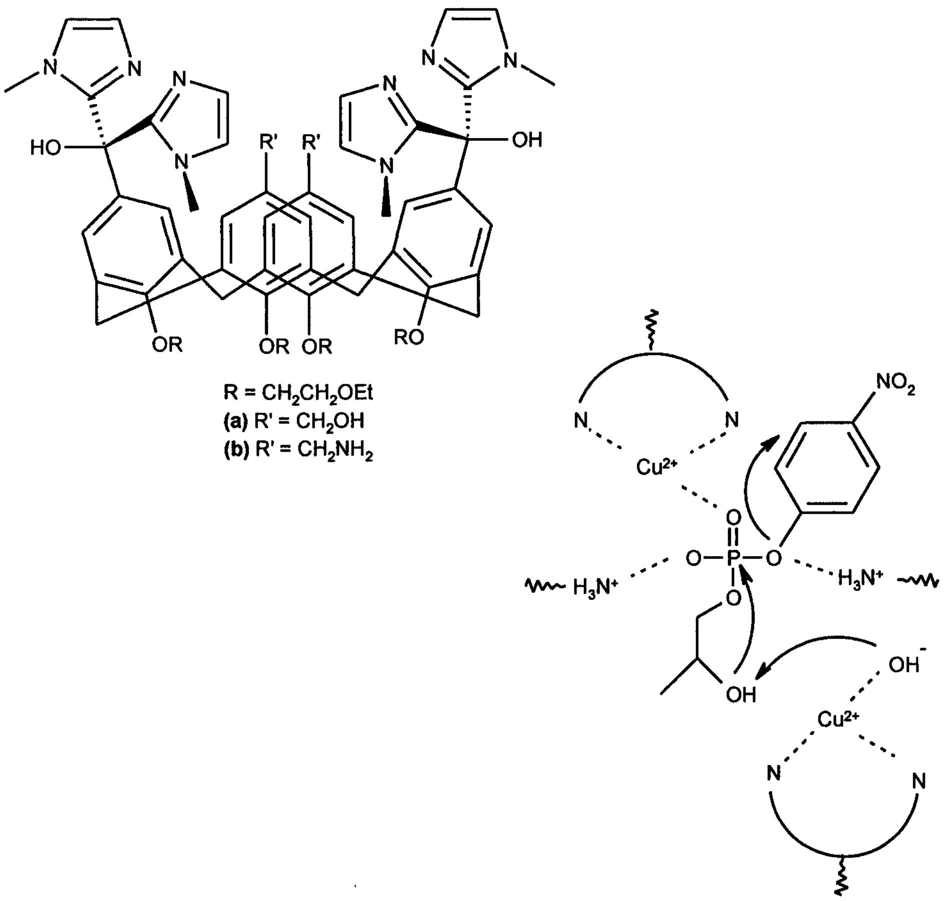

**Fig. 10:** Trans-esterification of DNA by calix[4]arenes bisimidazolyl-Cu(II) centers and two hydroxy methyl groups at the upper rim of the calix[4]arene were reported /10/ and their synthesis and the catalysis of the transesterification of HPNP were also described. The aminomethylcalix[4]arene derivative (Fig. 10, **b**) exhibits bifunctional catalysis in the intramolecular trans-esterification of the RNA model substrate HPNP. The substrate binding is due to cooperative action of one or both Cu(II) centers with one or two ammonium groups. Two ammonium groups and one of the Cu(II) centers may bind and activate the negatively charged phosphoryl group of the substrate. The remaining Cu(II) center can then contribute to the catalysis by the deprotonation of the substrate hydroxyl group via a bound hydroxide ion. The ammonium groups, further, stabilize the transition state and possibly the leaving group by electrostatic interactions and hydrogen bonding /10/. The hydroxy methyl calix[4]arene (Fig. 10, **a**) showed a catalytic activity that is in the same range as for calix[4]arene-Cu(II) complex (Fig. 8), i.e., a rate acceleration of a factor $6.9 \times 10^3$ over the uncatalyzed reaction. Dinuclear complex with two additional amino groups exhibits at pH 7.4 a rate acceleration of $3.1 \times 10^3$, but is not an efficient catalyst at pH 6.2. Hydroxy methyl calix[4]arene was found to be much less active at alkaline pH, indicated weak binding of the substrate to the catalyst. This might be caused by tightly bound Cu-hydroxides and steric hindrance by the hydroxymethyl groups.

Multinuclear metal arrays are ubiquitous components of metalloprotein active sites that participate in a number of important biological reactions. For example, tricopper sites have been identified in the multicopper oxidases /29/ and in particulate methane monooxygenase (pMMO), which use $O_2$ to couple the four-electron reduction of $O_2$ to $H_2O$ with the oxidation of substrate or to functionalize hydrocarbons, respectively. A tetracopper site has also been found that features ≤2 N-donors per metal in an important respiratory enzyme, nitrous oxide reductase /30/. Higher nuclearity species with three or four bound metal ions are less common and they also feature ligands comprising bidentate donors that would be expected to afford enhanced reactivity at the metal centers due to the greater accessibility of lower cordination numbers.

Tolman *et al.* /31/ developed a new set of multidentate ligands, which incorporate ≥3 bidentate N-donors that are covalently linked to and preorganized by the calix[4]arene framework for metal complexation. These ligands were synthesised with the aim of binding metal ions and exploring the chemistry of the resulting complexes, with the ultimate objective being to understand the reactivity, structures, and spectroscopic properties of multinuclear metal arrays found in Nature.

### 2.3. Acyltransferase enzyme mimic

Several binding interactions working in cooperation generate much greater flexibility and specificity in receptors. Hence, cooperativity of functional groups is important for the catalytic properties of the supramolecular enzyme mimic. In particular, imidazole moieties were often used as an acid-base couple or nucleophile, which can enhance hydrolytic processes or aldol type condensation reactions. Calix[4]arenes, which bear imidazole groups at different positions were reported recently by Schatz *et al.* /32/ as metal free enzyme mimics with trans-acyltransferase activity. Attachment of one nucleophilic group onto the upper rim resulted in an increase of the initial reaction rate. The macrocyclic skeleton improved the hydrolysis by 13% compared with the non-macrocyclic catalyst and by 52% towards the blank hydrolysis. Diimidazole calix[4]arene bearing the catalytic groups in a distal arrangement doubled the initial reaction rate indicating some kind of cooperativity of the catalytic sites. At pH 6.3, 50 % of the imidazole units are protonated and both imidazole groups can act as acid-base catalysts for the ester hydrolysis in aqueous solution. Calix[4]arenes possess enough flexibility to adopt a conformation suitable for the productive interaction with the substrate(*p*-nitrophenyl benzoate), to get it hydrolysed.

### 2.4 Ribonuclease enzyme mimic

The lanthanide ion based macrocyclic complexes mimic the hydrophobic nature of ribonucleases, where the lanthanide ions induce the formation of a hydrophobic cavity for glycine based macrocyclic compound, giving rise to a large order of magnitude enhancement in the hydrolytic cleavage of mRNA model compound HPNP /33/. A Zn(II) neocuproine based artificial rebonuclease has been constructed for the sequence-selective transesterification of RNA /34/.

Shinkai *et al.* /35/ reported the first totally man-made mimic for the regioselective catalysis of ribonuclease. The 2', 3'- cyclic phosphates of cytidine, uridine, adenosine and guanosine ( C>p, U>p, A>p & G>p) were hydrolysed in the presence of water soluble calix[n]arene–4–sulfonates and regioselective cleavage of ribonucleoside 2', 3' –cyclic phosphate was achieved successfully in acidic media by a nonenzymatic system, whereas the regioselective catalysis by cyclodextrins was effective only in neutral and alkaline media. Marginal regioselective catalysis was shown by calix[6]arene and calix[8]arene, which may be due to rapid exchange between cone, partial–cone, alternate and other conformations, while regioselective P – O(2') cleavage of C>p to cytidine 3' – phosphate was successfully achieved by calix[4]arene as catalyst. Both the regioselectivity and the reaction rate was found to increase with increasing concentration of calix[4]arene, exhibiting gradual saturation at large concentration of the calixarene. It was also noted that the cyclic structure of calix[4]arene is required for the regioselective catalysis as the regioselectivity was not increased by the monomeric analog 4–hydroxy benzenesulfonic acid. The cytosine residue of C>p is positively charged, whereas calix[4]arene is negatively charged (one of the hydroxyl group is negatively charged). The adenine residue of A>p and the guanine residue of G>p are also mostly protonated. Electrostatic interaction was found between calix[4]arene and the substrate was found to be essential for the catalysis.

### 2.5. ATPase enzyme mimic

Nucleotide polyphosphates, particularly adenosine tri- di- and monophosphates (ATP, ADP and AMP), are integral parts of the energy cycle for a vast range of biological processes, such as photosynthetic phosphorylation, oxidative phosphorylation, muscle contraction, and active transport. ATP provides the energy needed for the maintenance of cell transmembrane electric potentials. This energy comes from a class of enzymes termed ATPas. The function of ATPas is simply to hydrolysise the terminal phosphate residue of the triphosphate tail of ATP to yield ADP and inorganic phosphate ($H_2PO_4^-$, Pi). his process releases about 35 kJ $mol^{-1}$ of enengy and results in temporary phosphorylation of the enzyme.

An example of supramolecular catalysis showed a remarkable effect of supramolecular interaction on the catalysis of calix[4]arene (Fig. 11) on hydrolysis of ATP /36/. The hydroxides at the lower rim were found to cause strong intermolecular hydrogen bonding with guest molecules, using laser photolysis and pulse radiolysis. This electrostatic interaction between calixarene and substrate was suggested as essentiality for the

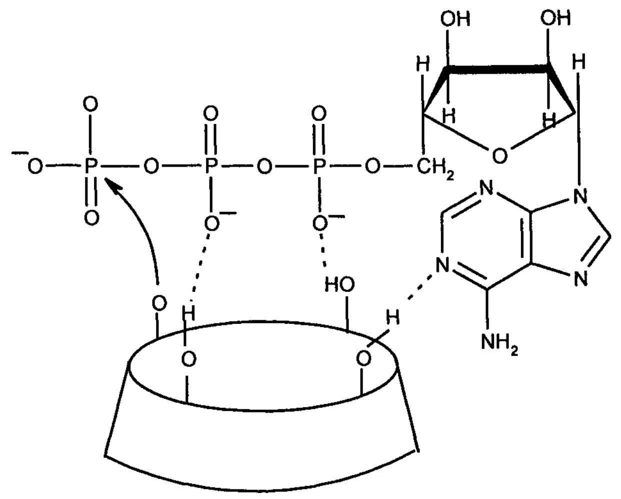

**Fig. 11:** The proposed structure of the supramolecular complex calix[4]arene with ATP

catalysis. The hydrolysis of ATP in pure aqueous solution was found to be slow and acceleration in the speed was noted after the addition of water-soluble calix[4]arene into the solution.

### 2.6. Aldolase enzyme mimic

The condensation of dihydroxy acetone phosphate(DHAP) to glyceraldehyde phosphate (GAP) is catalyzed by the aldolase enzyme to form fructose-1,6-diphosphate. A calix[4]arene derivative was proposed, which might serve as an aldolase model which holds a molecule of metal atom co-ordinated dihydroxy acetone phosphate in such a fashion that the keto group is proximate to amino function (reacting to Schiff base formation) and the α-hydrogens of the hydroxy methylene group are proximate to a carboxylate function (leading to a proton abstraction and formation of a carbanion as a charge separated enamine). Condensation with an aldehyde (glyceraldehyde phosphate in the biological reaction) can then rapidly occur at the exposed, unhindered face of the carbanion to yield the aldol product. In a similar way, numerous other enzyme mimic systems can be envisaged in which appropriate functional groups are arrayed around the cavities provided by calixarenes either in the 'cone' or 'partial cone' conformation /3/.

### 2.7. Heme mimics

Heme is an iron containing tetrapyrrole macrocycle. In heme proteins, the iron atom is either five or six coordinated. The fifth position is occupied by a so-called proximal iron ligand. This ligand may be a sulfur ion from cysteine molecule (cytochrome P-450 and chloroperoxidase) or a nitrogen atom of imidazole from histidine molecule as in secondary amine monooxygenase, catalase, peroxidase and $O_2$ carriers (hemoglobin and myoglobin). The sixth (distal) ligand may be exogeneous or supplied by protein (dioxygen, water peroxide etc.). Heme proteins carry out a number of various reactions including reversible dioxygen co-ordination (heamoglobin and myoglobin), one electron oxidation of organic substances (peroxidases), two electron oxidation of organic substances (cytochrome 450, secondary amine monooxygenase and diamine oxidase) and halogination (chloroperoxidase and thyroid peroxidase). Heme mimics have been developed and the structural and physical measurements obtained from simple model systems gave invaluable information about the mechanism of cooperativity in the binding of small molecules to hemoglobin and other heme containing systems such as peroxidases. The structural changes which occur at the heme center upon the binding of dioxygen, carbon monoxide, and alkyl cynanides to hemoglobins can also be understood using porphyrin containing models /37/. Previous studies of heme active site structures show that the primary difference is the length of the iron proximal histidine bond, which can be controlled by the degree of hydrogen bonding to this histidine. Great efforts to mimic these functions with synthetic analogues have been made for more than two decades. The peroxidase models resulted in several catalytic systems capable of a large range of oxidative transformations. Most of these model systems modified the porphyrin ring covalently by directly binding auxiliary elements that control and facilitate reactivity; for example, electron donating or withdrawing substituents. A biomimetic approach can be designed by attempting to keep the porphyrin ring system unaltered, as close as possible to its native form and introducing all modifications at or close to the axial coordination sites. Such a model system is less demanding synthetically and provides an easy way to probe the effect of a single structure modification. A two-component system (a bisimidazolyl ligand and an iron-porphyrin) was reported. Introduction of asymmetry, by attaching different imidazoles as head groups, led to the formation of two axial bonds of different length. Addition of hydrogen bonds to one of the imidazoles in an advanced model increased these differentiation and expanded the porphyrin ring. These biomimetic complexes were found to be almost identical in structure to peroxidase active sites /37/.

In the quest of developing non-porphyrin heme mimic, calixarene derived heme-mimics have been reported. Cram's cavitand (Fig. 12) /38/ showed oxygen affinity. In an effort to make another non-porphyrin heme-mimic Gutsche and Nam investigated the metal complexation behaviour of *p*-(2-amino-ethyl)calix[4]arene (Fig. 13). One of the reasons for undertaking the synthesis was to explore its complexing abilities with metal ions such as $Fe^{2+}$ and its potential for acting as an oxygen carrier. The purpose was that it would form an octahedral complex, in which the amino groups occupy four equatorial sites around a metal

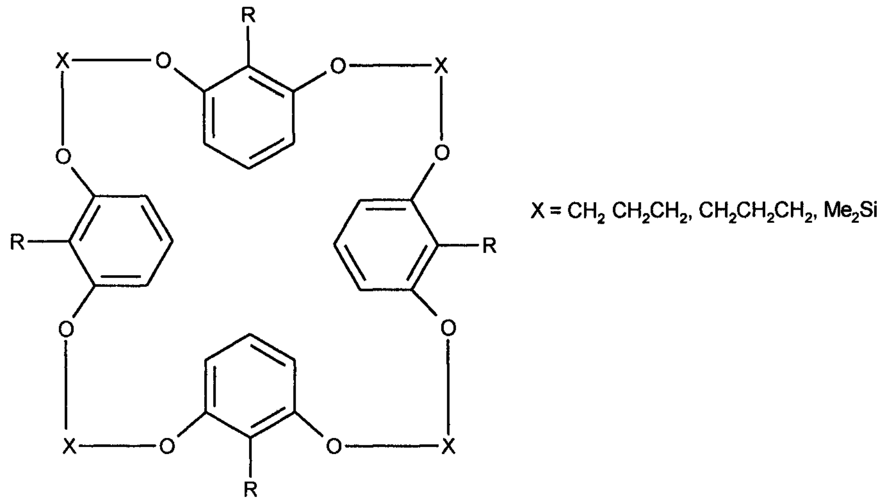

**Fig. 12:** Cram's cavitand

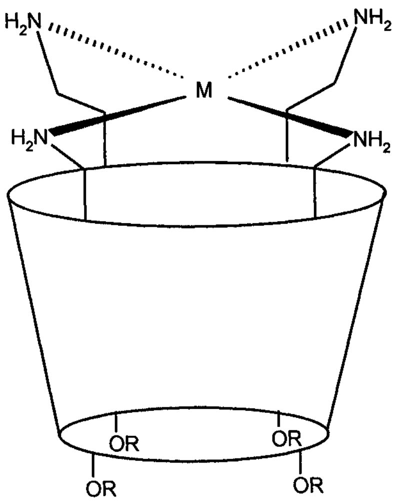

**Fig. 13:** *p*-(2-Aminoethyl) calix[4]arene ion, an external ligand such as an amine occupies a fifth (apical) site leaving the sixth site (apical) pointing into the cavity of the calixarenes and accessible only to molecules which are small enough to pass through the annulus at the lower rim of the calixarene /39/.

### 2.8. Carbonic anhydrase enzyme mimic

Transition metal complexing agents bearing active amino acid subunits were synthesized /40/ to build lipophilic ligands containing heterocyclic chelating arm preorganized on a calix[4]arene platform, which could mimic the activity of some metalloenzymes. Calix[4]arene podands bearing two or four histidine or glycine groups at the upper rim were synthesized to prepare a carbonic anhydrase biomimetic model. Complexation properties of cobalt(II) chloride were investigated and it was found that the complexation of Co(II) with the glycyl podands was unsuccessful but histidyl analogues formed blue-colored species.

### 3. BIOMIMETISM BASED ON STRUCTURE

The task of mimicking the enzymatic reactions in the laboratory is difficult. The main reason was the lack of knowledge of enzymatic mechanisms and the singularity of chemical reactions in the biological systems. Various methods now exist to determine their functioning and it is becoming ever more clear. Because of the high molecular weight of enzymes reaching tens and hundreds of thousands, determination of structure is far from being a simple task. But, by structural mimics, we obtain the coordinates of all the atoms, including those constituting an enzyme active center and its closest surrounding. From this analysis, it can be concluded that the mechanism of enzymatic catalysis is analogous to the mechanism of the usual chemistry. The main difference is the much more complex and perfect organization of the interacting molecules in the enzyme active center, which leads to the qualitative difference in the chemistry of living nature, as compared with ordinary chemistry. A key step that needs to be completed in metalloenzyme mimics is to design a ligand that can reproduce the biocoordination sphere of the metal ion. A number of research groups have designed models aimed at reproducing the biological coordination core of the metal ion.

### 3.1. Importance of Cu(I) and Cu(II) in biological systems

Copper is a very important metal ion in biological systems, playing a very diverse role in living organisms /42/. It is involved in biochemical oxido-reduction processes, in electron transfers, as well as in reversible binding of small gaseous molecules such as $O_2$ (hemocyanine) or the plant hormone ethylene. Because the active form in these systems involves Cu(I), the co-ordinate Cu(I) compounds are essential to the understanding of the metal reactivity. Spectroscopic probes for the Cu(I) center are lacking in native proteins and hence it becomes important to study its coordination chemistry with small artificial ligands /43/. Binding characterisitics of Cu(II) are equally important, as copper enzymes are involved in redox biological processes.

### 3.2. Calixarene based $Cu^I$ and $Cu^{II}$ complexes as structural models

Mononuclear copper enzymes constitute an important class of proteins /42,44/. Very few chemical systems allow the specific chemistry at a monocopper site to be modelled. A supramolecular model of the mononuclear cuprous sites was described. Advantage has been taken of the conic shape of a calix[6]arene appropriately functionalized with three pyridine groups. The role of the pyridine group is to provide an $N_3$ donor set for the copper ion, which allows the formation of a stable mononuclear transition metal complex and in the construction of a receptacle, which interacts with an external molecule. This system mimics the imidazole residues, which co-ordinate the type II cuprous ions in enzymes such as dopamine- β- hydroxylase, peptidyl glycine, α-amidating mono oxygenases, copper-amine oxidases and nitrate reductases /44/. The role of the calix[6]arene is to protect any undesired interaction with another metal center to facilitate the approach of an organic molecule and finally to operate a means of selection based on its size and nature /45/. To evaluate the importance of the role of the cavity, a slight modification was done in ligand-a shown in Fig. 14, where three out of the six calixarene t- Bu substituents were removed /46/.

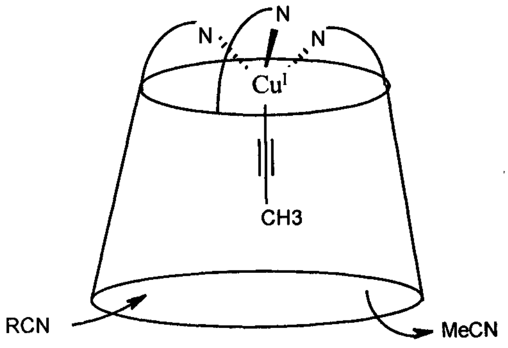

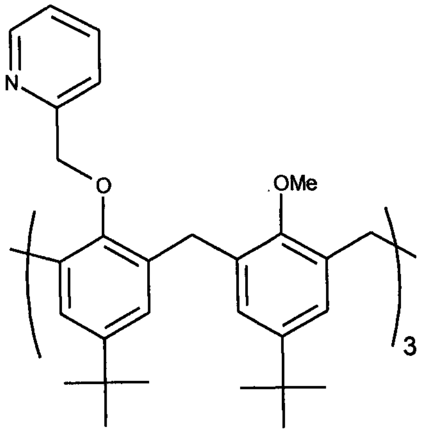

(a)

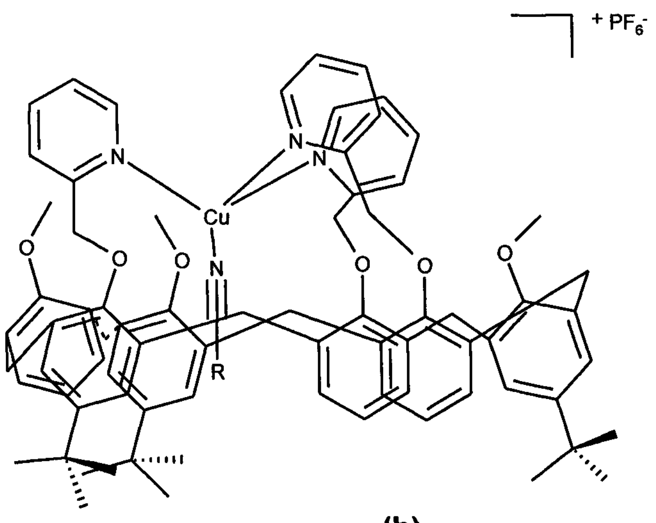

(b)

**Fig. 14:** Calixarene based Cu(II) complexes as models for mono copper sites in enzymes

The partially debutylated ligand-**b** offers a larger pocket with a wider opening than ligand -**a**. The comparative binding of different nitriles to these calixarene based mono-nuclear Cu(I) centers were studied by $^1$H NMR Spectroscopy and was found that the recognition pattern for the cuprous complexes for nitrilo ligands was inverted as the affinity of host-**b** compared to that of host-**a** for PhCN Vs. MeCN gained 3 orders of magnitude. Removal of three calixarene-t-Bu groups led to a 100- fold acceleration of the MeCN exchange rate. The calixarenes have a strong tendency to adopt a conformation well suited to optimize the interaction of their aromatic moieties with other groups. So the calixarene conformation acts as a selective molecular funnel for neutral molecules. The biomimetic complexes derived from calixarenes maintain a high degree of flexibility which is known to be essential in natural system, in particular for fast substrate access and product departure. Hence, these supramolecular systems provided a rare and selective model for the hydrophobic substrate channel giving access to metalloenzyme active site. In an another example, calix[4]arene (Fig. 15) was used as a platform for the stabilization of a two co-ordinate Cu(I) complex with two biomimetic imidazolyl pendant arms. This complex appeared to be extremely resistant to $O_2$ oxidation and was stable in air for weeks, even in potentially coordinating or protic solvents /43/.

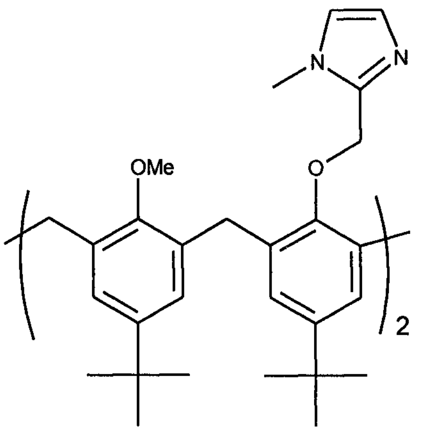

**Fig. 15:** Calix[4]arene with biomimetic pendand arms

The presence of two imidazole rings rather than two pyridine rings provided enhanced stability of the copper(I) complex towards oxidation. This may be due to electronic factors and may be explained by an increased orbital overlap between the imidazole-nitrogen atom and copper(I) that stabilizes the two coordinate form for the imidazole ligated species compared to the pyridine. Indeed, the copper(I) complex derived from the corresponding pyridine-based calix[4]arene ligand was extremely sensitive to $O_2$ and underwent auto-oxidation in air within seconds. This emphasizes the importance of preorganization of the metal binding site as one of the key features that allows the control of the $Cu^I$ reactivity. This may be reminiscent of biological systems, which either utilize copper(I) to bind or activate a small molecule or stabilize it as in copper chaperones which transport or deliver $Cu^I$ to other proteins in the cells /43/.

Reinaud *et al.* /46/ described four novel calix[6]arene based cuprous complexes, which present a biomimetic tris (imidazole) co-ordination core, associated with a hydrophobic cavity that wraps the apical binding site. The strong conformational coupling between the metal center and the host structure provides a supramolecular control to the system. The vibrational spectra of biomimetic-Copper(I)- CO complexes were revealed to be a sensitive gauge of this supramolecular behaviour, similar to the copper proteins in which allosteric effects are common.

The synthesis of biomimetic complexes that are soluble and stable under physiological conditions is an important goal. In the course of modeling type II active sites of copper enzymes, the synthesis of the first water-soluble biomimetic cuprous calix[6]arene complex (Fig. 16) was described /49/, which allows the formation of a $N_3Cu(I)$ complex with a fourth binding site that is accessible for an exogeneous ligand, with a strong affinity for the metal ion towards CO. The presence of CO was confirmed by an absorption peak at 2075 $cm^{-1}$ in IR spectrum.

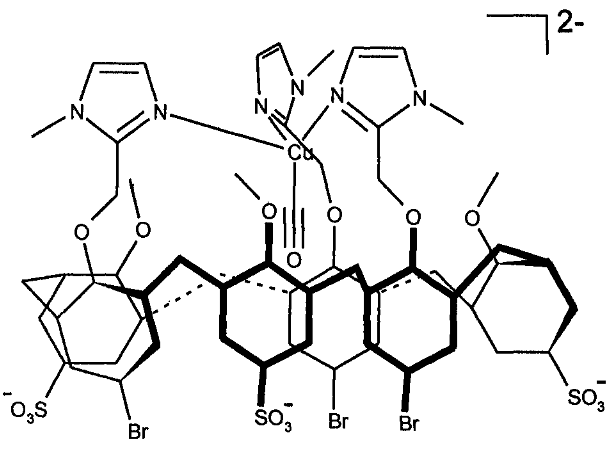

**Fig. 16:** Biomimetic cuprous calix[6]arene

This cuprous complex is resistant to disproportionation and to air oxidation. It also shows that the co-ordinating environment provided by three imidazole moieties, which mimics tris(histidine) binding site in enzymes, is indeed capable of stabilizing a cuprous center in water. Although, hexasulfonated calix[6]arene derivatives have been described as highly flexible molecules with a preferred 1,2,3- alternate conformation /44/, the rigidity was achieved of the hosts from the binding of a metal cation and the cone conformation is favored by the relatively smaller electrostatic repulsion on the large rim bearing only three sulfonates /49/.

As copper enzymes are involved in redox processes, therefore it becomes very important to explore the coordination of Cu(II) to these calixarene-based ligands. So, the structure and host-guest behavior of the first cupric complexes was described.

After the addition of the individual nitrile to a dichloromethane solution of complex, it was observed that there is the distortion of the tetrahedral geometry toward trigonal bipyramidal due to an interaction between MeCN and the metal center. The addition of propionitrile or allylnitrile also induced significant changes to EPR and visible spectra, whereas more bulky benzylnitrile was ineffective. This suggests that the complex possesses an exchangable coordination site that selectively interacts with small molecules. It was concluded that a second aquo ligand that is buried inside the hydrophobic cavity and can be selectively exchanged for lipophilic molecules. The calixarene hydrophobic pocket protects the metal center against dimerization and simultaneously acts as a molecular funnel for organic molecules /50/.

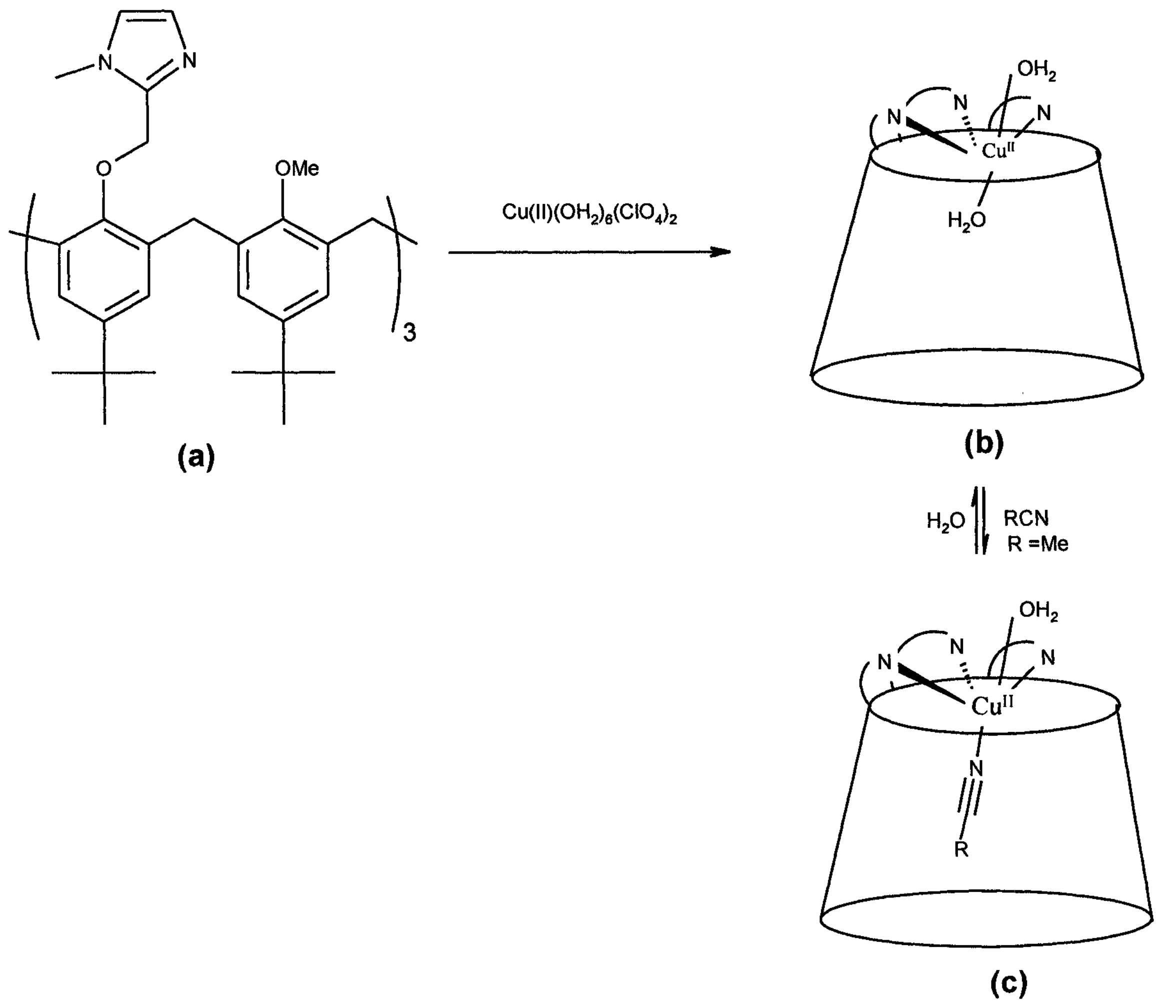

**Fig. 17:** Cupric complex of calix[6]arene as a molecular funnel

### 3.3. Importance of $Zn^{II}$ in biological systems

Zinc is a constituent of more than 300 enzymes. The active sites of zinc enzymes feature a zinc center attached to the protein backbone by three or four amino acid residues, the nature of which influences the specific function of the enzyme. Different zinc enzymes utilize different amino acid residues at the active site and hence the chemistry of zinc is modulated by its coordination environment. Zinc(II) ion is a biologically essential element. Recognition of its importance has been increasing as more enzymes containing zinc(II) in their active site have been discovered /51/. Bioinorganic chemistry of zinc enzymes that features tripodal ligands has been described /52/. These enzymes are mainly involved in hydrolytic processes but also catalyze the reversible oxidation of alcohols. Hence, the enzyme active center interacts with molecules with various functionalization such as amides, carboxylic acids, amines, alcohols, aldehydes, etc. Carbonic anhydride, matrix metalloproteases, bacillus cereus β-lactamase II and 6 pyruvoyl tetra hydropterin synthase all possess a common feature in their active site: a mononuclear zinc center coordinated by three histidines and a water molecule. Several research groups have structurally modelled the coordination environment of the zinc center using a variety of lignads /53/, such as macrocyclic triamines, tris(imidazolyl) – phosphines or the anionic tris(pyrazolyl) borates /54/. All these models focused on Zn-hydroxide complexes.

### 3.4. Calixarene based $Zn^{II}$ complexes as structural models

Reinaud and coworkers /55/ extended their work to a supramolecular system (Fig. 18) which possesses a tris(histidine) coordination core hereby mimicking the active center of Zinc proteins. This calix[6]arene based system is highly versatile since it allows the tuning of both the steric hindrance and the electronic properties due to the N donor set. Upon co-ordination to a $Zn^{2+}$ ion, this ligand gave rise to a remarkable biomimetic dicationic zinc-aqua complex. The highly acidic $Zn^{2+}$ center was constrained in a tetrahedral environment with a labile site oriented toward the inside of the calix[6]arene structure.

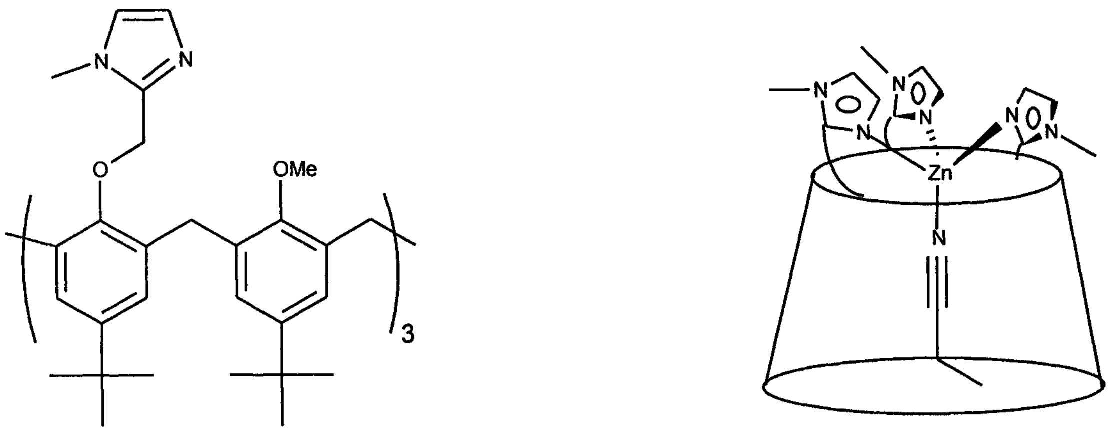

**Fig. 18:** Novel biomimetic calix[6]arene based Zn-complexes

The hydrophobic pocket acted as a selective molecular funnel for the neutral molecules. $^{1}$H-NMR spectroscopy studies showed the easy exchange of the aqua ligand for amines, alcohols, amides or nitriles. This very sensitive but stable receptor is selective, based on both the chemical nature of the guests and their size and shape. With primary amines, coordination to the metal center was stoichiometric and quantitative. For acetamide, ethanol and DMSO, the binding to $Zn^{2+}$ was ~ 20-30 times stronger than for water. DMF was also an excellent guest, that is, bound more strongly to the metal center than nitriles. Coordination to even weaker donor molecules, such as propionaldehyde and acetic acid, could also be detected. In these cases, however, the aqua species remained by far the dominant one. Finally, ether, THF, acetone did not yield any detectable coordinated species. Secondary, tertiary and aromatic amines did not yield any detectable coordinated species. Coordination of 2-propanol was 30 times weaker than that of 1-propanol. However, ethanol was more strongly coordinated than 1-propanol. Benzo- and benzyl-nitrile appeared too sterically encumbered to yield a stable adduct. In all these respects, this system resembles the Michaelis complex of zinc enzymes and may lead to the design of selective chemical sensing agents /56/. The same working group also presented two novel adducts, one with ethanol and the other with formamide. Their x-ray structures revealed that hydrogen bonding and CH/π interactions between the calix[6]arene host and the guests play an important role in stabilizing the complexes. These provide the first examples of stable four co-ordinated Zn(II) dicationic complexes presenting a terminal aliphatic alcohol or amide- ligand. Both compounds are strongly relevant to the enzyme/substrate complexes, in mononuclear zinc biological systems. These adducts are interesting structural models for the species involved in the catalytic cycle of zinc-peptidases or LADH as these enzymes present similar stabilizing interactions. The new data also suggest that a sulfur-rich co-ordination core as in LADH is not a specific requirement for the stabilization of tetrahedral zinc alcohol complexes but might rather be necessary for substrate activation towards hydride transfer.

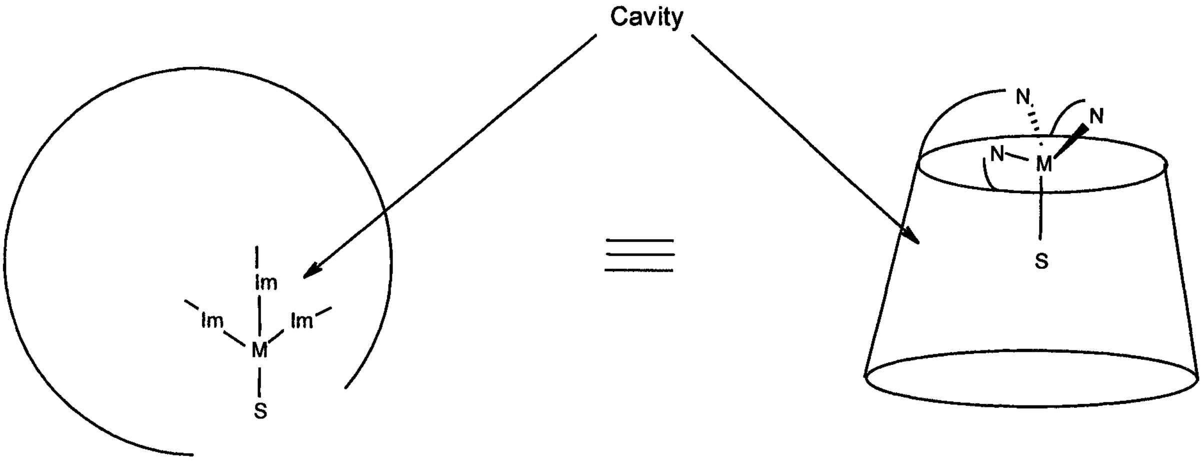

**Fig. 19:** A "funnel complex" modelling the mononuclear active site of enzymes

Tridentate N ligands containing tertiary amines, pyrazoles or benzimidazole groups were synthesized from t-Bu-calix[6]arene. The comparative ability of the whole series to stabilize a tetrahedral zinc dication was studied in a biomimetic neutral environment. The study showed that benzimidazole and pyrazole are also appropriate N donors for the stabilization of such complexes along with imidazole-based ligands. The calixarene structure is constrained in a flattened cone conformation thereby providing a hydrophobic cavity, which envelopes the exogeneous ligand, MeCN. It was found that the calixarene presenting three pyridine groups did not give rise to a well defined complex under stoichiometric conditions. Hence, pyridine appeared to be a N donor not good enough for the stabilization of tetrahedral dicationic zinc complexes. This is in contrast to its good capacity at stabilizing $Cu^{I}$, and may be due to its softness /57/.

### 4. BIOMIMETISM BASED ON MOLECULAR RECOGNITION AND IMPORTANCE OF HYDROGEN BONDING

Molecular recognition, a process involving both binding and selection of substrates by a given receptor molecule, as well as possibly a specific function, has inspired many organic chemists to design artificial receptors. The recognition of polar organic molecules of biological interest such as carbohydrates /58/ and amino acids /59/ is a topic of current interest in supramolecular chemistry. Macrocyclic receptors are particularly attractive in this field, which are characterized by the simultaneous presence of different binding groups, e.g. polar groups and hydrophobic cavities, which cooperate in recognition process. Calix[4]arenes functionalized at the upper rim were synthesized with carbohydrates /60/, (glyco-calixarene), amino acids /61/ (N-linked peptido calix[4]arene) and (thio) urea /62/ units which represent examples of such hybrid receptors developed for the complexation of sugars, amino acids, and anions.

Reported data reveal that the complexation of the α-amino acids occurred by insertion of the aromatic or aliphatic apolar group(R) into the calixarene cavity. L-Alanine is not complexed by any hosts, owing to the small size of the R methyl group; the inclusion of the L-Ala would also involve the encapsulation of the amino acid charged species, inside the hydrophobic cavity. No inclusion is observed for L-Tyr either, very likely owing to the polarity of the R group.

Calixarene (Fig. 20, **c**) which lacks the sulphonate groups at the upper rim, does not complex any of the investigated amino acids, which proves the significance of charge assistance in the apolar binding of the guests inside the cavity of calixarene /64,65/. The most efficient receptors for the amino acid studied are compounds **a** and **b** (Fig. 20). Capabilities of the hosts **a-e** decrease from **a** to **e**, because of the $C_{2v}$ elongation of the cavity. Host **a** has a more spherical cavity, whereas in host **e**, substituents at the lower rim are diverging which results in the elongation of the cavity and hence less accessibility for the amino acids. In all the cases inclusion occurs via an aliphatic or aromatic apolar residue due to CH- π interactions (L-Val – and L-Leu) or to π – π interactions (L-Phe, L-His, L-Trp) /63/. These studies were extended to several small neutral organic molecules, i.e. methanol, ethanol, n-propanol, acetone, butanone and acetonitrile as guests using compounds **a-e**. The complexation of methanol by the hosts is disfavoured because inclusion of the small methyl group inside the hydrophobic cavity would lead to a partial inclusion of the polar OH group, which would cause the polar hydroxyl group to be less exposed to the polar solvent. Host-**e** could not include any of the guests due to the peculiar conformation of the host, which reduces the available space for complexation. Host-**b** was found to be more efficient for ethanol and n-propanol, whereas conformationally mobile compound-**a** showed a better binding for acetone and 2-butanone. These data showed that the

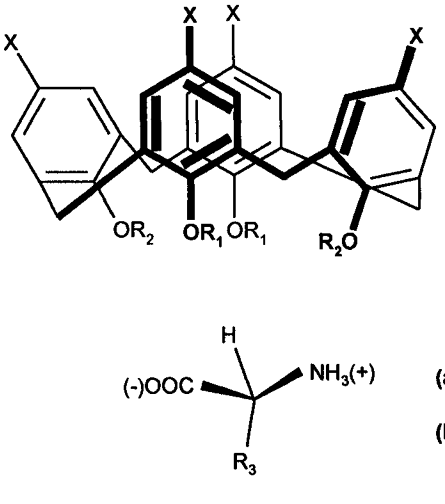

**(a)** $X = SO_3H$, $R_1 = R_2 = H$

**(b)** $X = SO_3H$, $R_1 = R_2 = CH_2COOH$

**(c)** $X = H$, $R_1 = R_2 = CH_2COOH$

**(d)** $X = SO_3H$, $R_1 = H$, $R_2 = CH_2CH_2OCH_2CH_3$

**(e)** $X = SO_3H$, $R_1 = R_2 = CH_2CH_2OCH_2CH_3$

**(a)** $R_3$ = (aliphatic)

L-Ala, L-Val, L-Leu

**(b)** $R_3$ = (aromatic)

L-Phe, L- Tyr, L-His, L-Trp

**Fig. 20:** Complexation of amino acids by calix[4]arene derivatives inclusion capabilities of the investigated hosts are strictly correlated with their conformational properties /66/.

Recognition of carboxylate anions by natural and synthetic hosts is a topic of current interest in bioorganic and supramolecular chemistry, because these species are involved in several molecular recognition phenomena of biological interest. The enantioselective recognition of chiral carboxylate is also an important goal because several pharmaceutical compounds possess this functional group. Very few examples are known where natural amino acids are used as binding units in biomimetic receptors for carboxylate recognition. The intrinsic lower H-bonding donor ability of peptide NH groups could be strengthened by the cooperative action of other weak non-covalent interactions (π/ π, CH/ π, etc.) and resulting host-guest binding could be enhanced. Several cone calix[4]arenes functionalized with L-alanine units at the upper rim were synthesized /61a/. One of these derivatives (Fig. 21) containing four amino acid moieties was water soluble at neutral pH.

Inclusion properties of the host (Fig. 21) with several guest molecules especially with chiral amino acids were investigated by $^1$H NMR titration experiments /67/. It was found that the inclusion of L-phenylalanine (L-Phe) or its methyl ester hydrochloride (L-Phe Me) does not take place into the calixarene cavity, because of its residual conformational mobility between two $C_{2v}$ conformations. So, two short di(ethylene glycol) units in proximal (1,2) positions at the lower rim of calix[4]arene to inhibit its residual conformation freedom blocking it in a rigid cone structure /68/.

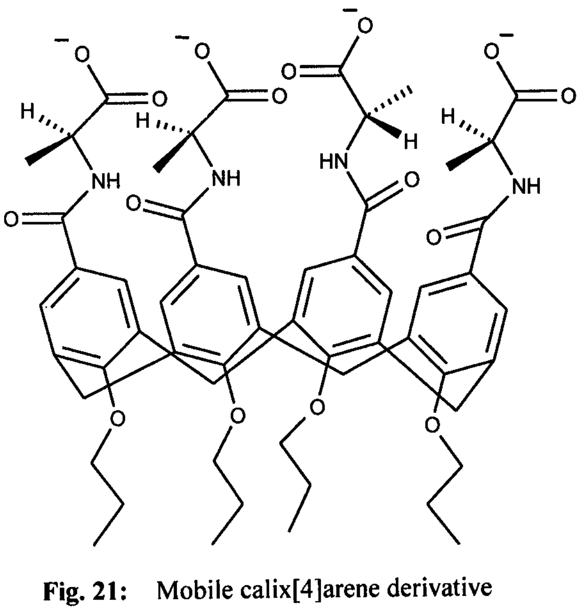

**Fig. 21:** Mobile calix[4]arene derivative

Complexation properties of the host (Fig. 22) towards quaternary ammonium salts, amino acids and their methyl ester hydrochlorides were evaluated /61b/ in $D_2O$ at pD=7.3 (phosphate buffer). The values for binding constants clearly indicated that the rigid cone peptidocalixarene (Fig. 22) was much more efficient than the mobile cone analogue (Fig. 21), in the complexation of all guests, which suggests the importance of the rigidity in the recognition process. Data also showed that amino acids are better complexed as methyl esters, rather than as zwitter ions, because better complementarity is achieved with the tetra anionic host (Fig.

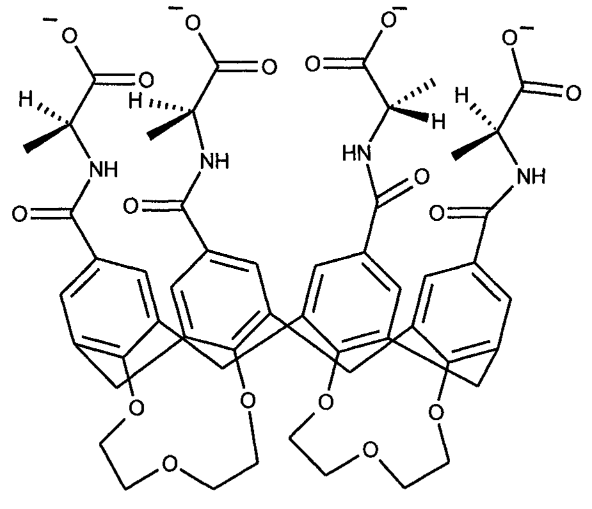

**Fig. 22:** Rigid cone peptidocalix[4]arenes

22) with substrates terminating in a positive charge only. Aromatic amino acids were more strongly complexed than the aliphatic ones and the highest association constant was observed for tryptophan. The interaction between the indole nucleus of Trp might be stronger than for the aromatic (Π- Π) or aliphatic (CH- Π ) groups of other amino acids. Very hydrophilic glycine and its Me ester also did not form inclusion complex with the host.

The recognition properties of C-linked peptidocalix[4]arenes (Fig. 24) were investigated in DMSO-$d_6$ by $^1$H NMR titration experiments /69/. No complexation was observed for linear ammonium cations, N-protected amino acids or carboxylic acid where the corresponding anionic forms were found to be complexed. The binding seemed to involve only the NH groups. The recognition behavior of peptidocalix[4]arene was reversed by changing the mode of attachment of the amino acid units to the calixarene platform, as compared to N-linked peptidocalix[4]arenes (Fig. 23), which complex carboxylic acids and ammonium cations but not anions in $CDCl_3$. The difunctionalized C-linked peptidocalix[4]arenes

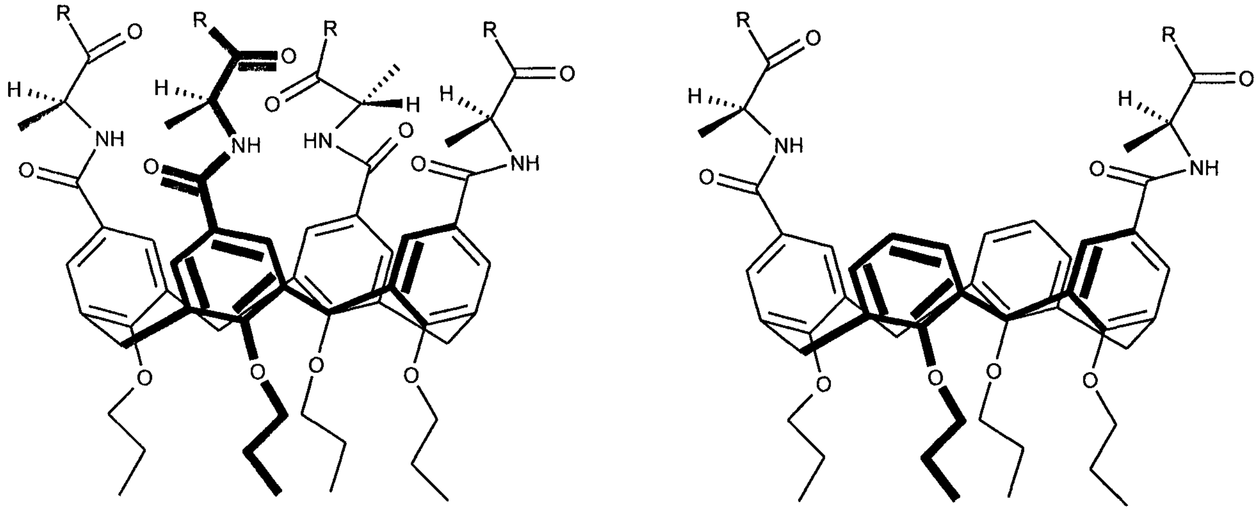

R = $OCH_3$, OH, $NHNH_2$, $NHCH(CH_3)COOCH_3$

**Fig. 23:** N-linked peptidocalix[4]arenes were found to form intramolecular hydrogen bonding, which stabilizes the pinched cone conformation with a closed cavity, whereas the N-linked peptidocalix[4]arenes showed a preference for the other pinched cone conformation, characterized by an open, cleft-like cavity. The tetrafunctionalized C-linked peptidocalix[4]arenes (Fig. 24) with four terminal amino groups, protonation of which leads to positively charged water soluble receptors, whereas the N-linked water soluble peptidocalix[4]arenes have negatively charged carboxylate end groups. These studies gave totally different interaction modes of the two classes of receptors with biologically important hosts such as enzymes, DNA, etc.

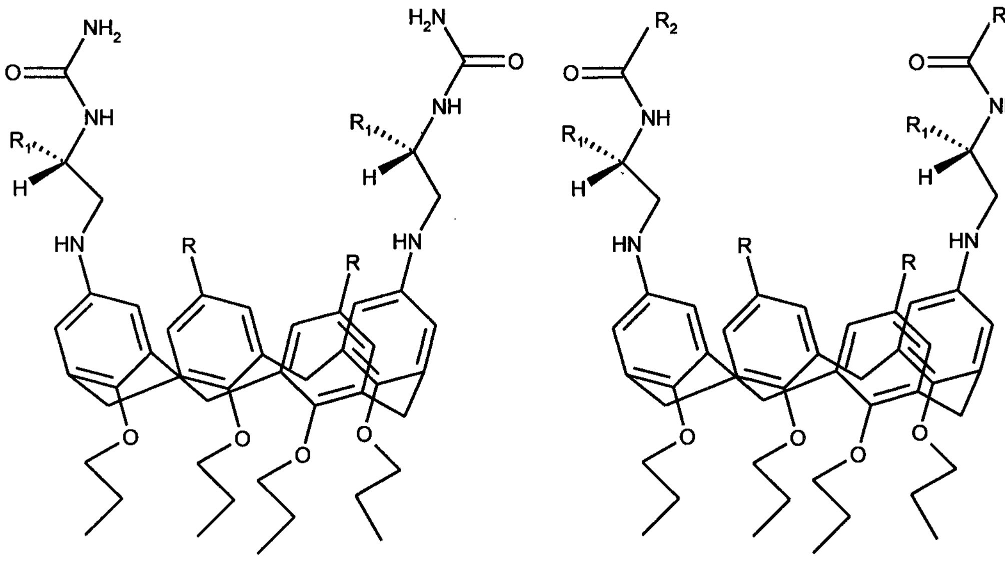

**(a)** $R_1 = CH_3$, R= $NHCOCH(CH_3)NH_2$

**(b)** $R_1$ = Bn, R = $NHCOCH(Bn)NH_2$

**(a)** $R_1 = CH_3$, $R_2 = CH_3$

R = $NHCOCH(CH_3)NHCOCH_3$

**(b)** $R_1$ = Bn, $R_2 = CH_3$

R = $NHCOCH(Bn)NHCOCH_3$

**(c)** $R_1 = CH_3$, $R_2$ = Ph

R = $NHCOCH(CH_3)NHCOCH_3$

**(d)** $R_1$ = Bn, $R_2$ = Ph

R = NHCOCH(Bn)NHCOPh

**(e)** $R_1 = CH_3$, $R_2 = CH_3$, R = H

**(f)** $R_1$ = Bn, $R_2 = CH_3$, R = H

**Fig. 24:** C-linked peptidocalix[4]arenes

Macrobicyclic peptidocalixarene (Fig. 25) was reported as a receptor for the D-alanyl-D-alanine terminal part of peptidoglycans. One interesting outcome of the study was that the *in vitro* behavior of peptido calixarene was similar to that of the vancomycin group antibiotics and its selective antimicrobial activity toward gram positive bacteria was only slightly inferior to that of vancomycin /70/.

Further, the binding affinity of vancomycin mimic with several guests were studied and correlated with observed biological activity. It was found that the host molecule being a vancomycin analogue, has a significant affinity to the ala – ala moiety, which is believed to be the action site of the vancomycin antibiotics /71/.

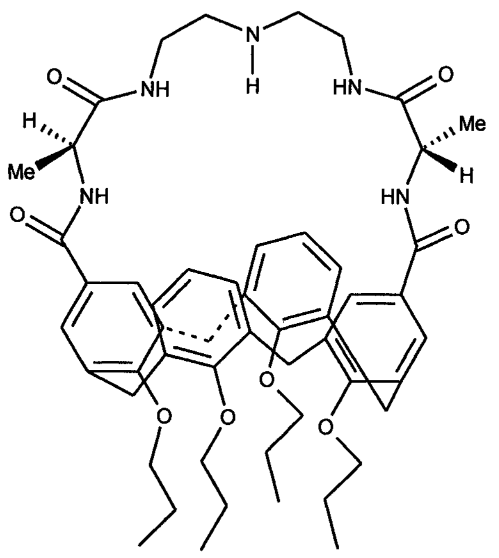

**Fig. 25:** A vancomycin mimic

Both the N-linked and C-linked (Fig. 26. **a** & **b**) cleft like peptidocalix[4]arene are very poor receptors in the recognition of ions and polar organic molecules, mainly because of their residual conformational flexibility, which in the case of C-linked compounds, leads to extensive intramolecular H bonding (e.g. Fig. 26, **b**). To reduce the conformational flexibility, C-linked peptidocalix[4]arenes were extended with a cap with suitable aromatic units and to explore the host properties of macrobicyclic receptors (Fig. 27), in particular toward anionic guest species.

It was found that these new ligands form strong complexes in acetone-d6 with carboxylate anions and are selective receptors for benzoate anion, which is able to interact with the hosts through the H-bonding and π-π stacking. It was concluded that the residual conformational mobility of cone calix[4]arenes is blocked by bridging it with 2,6- diacylpyridine or isophthaloyl moieties, which results in more preorganized receptors with enhanced efficiency. Selectivity toward aromatic carboxylates and especially toward benzoate is observed due to π-π stacking interactions with the pyridine moiety and/or a calix[4]arene aromatic nucleus which act in addition to H bonding with amide NH bonds /71,72/.

Amino acids exist as strongly solvated zwitter ions in neutral aqueous solutions. Their extraction and transport through the lipophilic membranes is very difficult. Although a number of synthetic receptors binding either ammonium or carboxylate moieties have been reported /73/, very few examples of zwitter ionic amino acids transport are known /74,75/.

α-Amino phosphonates possess an array of potential binding sites for both ammonium (Phosphoryl group, nitrogen lone pair) and carboxylate (N-H bond) moieties. Antipin *et al.* described the transport of amino acids through a liquid membrane supported on a polymer film induced by α-Amino phosphonates /76/.

The calix[4]arene is a rigid scaffold and provides lipophilicity to the whole molecule. 1,3-diaminocalix[4]arenes were selected as starting material because they have a tweezer like structure and are likely to combine the appropriate binding mode with the lack of steric hindrance.

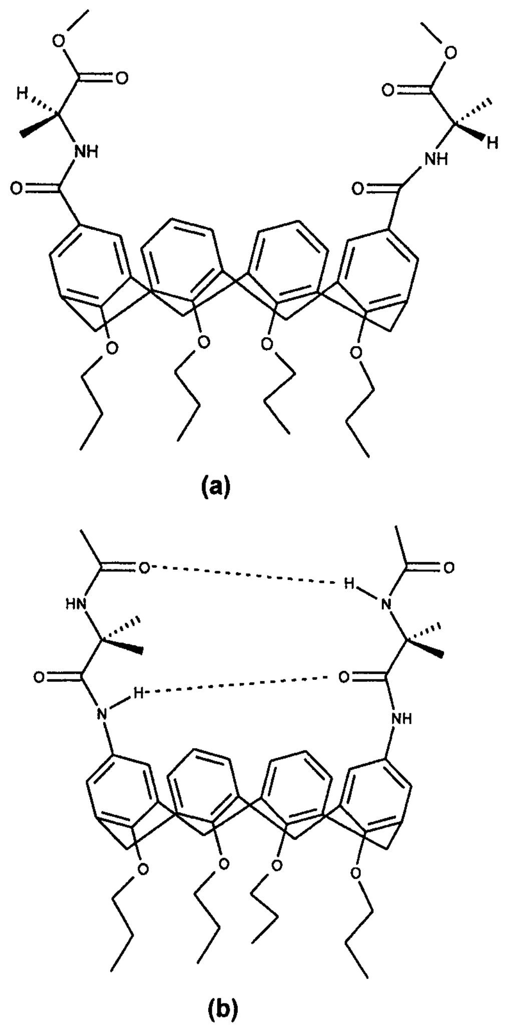

**Fig. 26:** Peptide calix[4]arenes

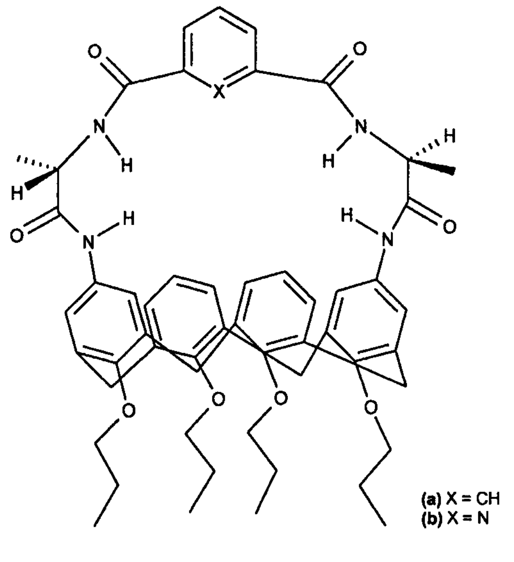

**Fig. 27:** Capped calix[4]arenes as biomimetic receptors

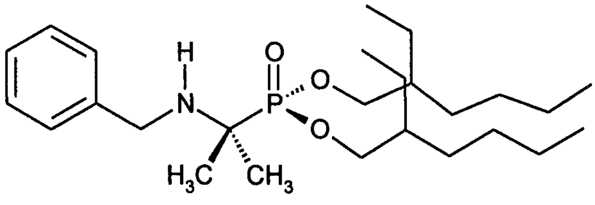

**Fig. 28:** Linear $\alpha$-aminophosphonate

Receptors shown in Fig. 29 were used /76/ as carriers for the transport of zwitter ionic amino acids through a supported liquid membrane composed of a porous polymeric support (Millipore Type FA) impregnated with $10^{-1}$ M carrier in *o*-nitrophenyl -*n*-octyl ether (NPOE). The results showed that hosts (Fig. 29, **b** and **d**) transport the amino acids with essentially the same rate as hosts (Fig. 29, **a** and **c**), which showed that the nature of the alkyl group does not play a significant role to the efficiency of the membrane transport. The attachment of amino phosphonate moieties to the lower and upper rim of the calix[4]arene lead to the different changes in the rate and selectivity of amino acid transport. With the exception of histidine, the transport rate of studied amino acids with lower rim functionalized calixarene (Fig. 29, **a**) and linear aminophosphonate was not having significant difference. It was suggested that the interior of the calixarene presents a polar microenvironment complementary to the zwitter ionic form of histidine. Besides carboxylate and ammonium groups, the imidazole side chain of histidine possess proton donor and proton acceptor center which interacts with amino phosphonate units of the calixarene.

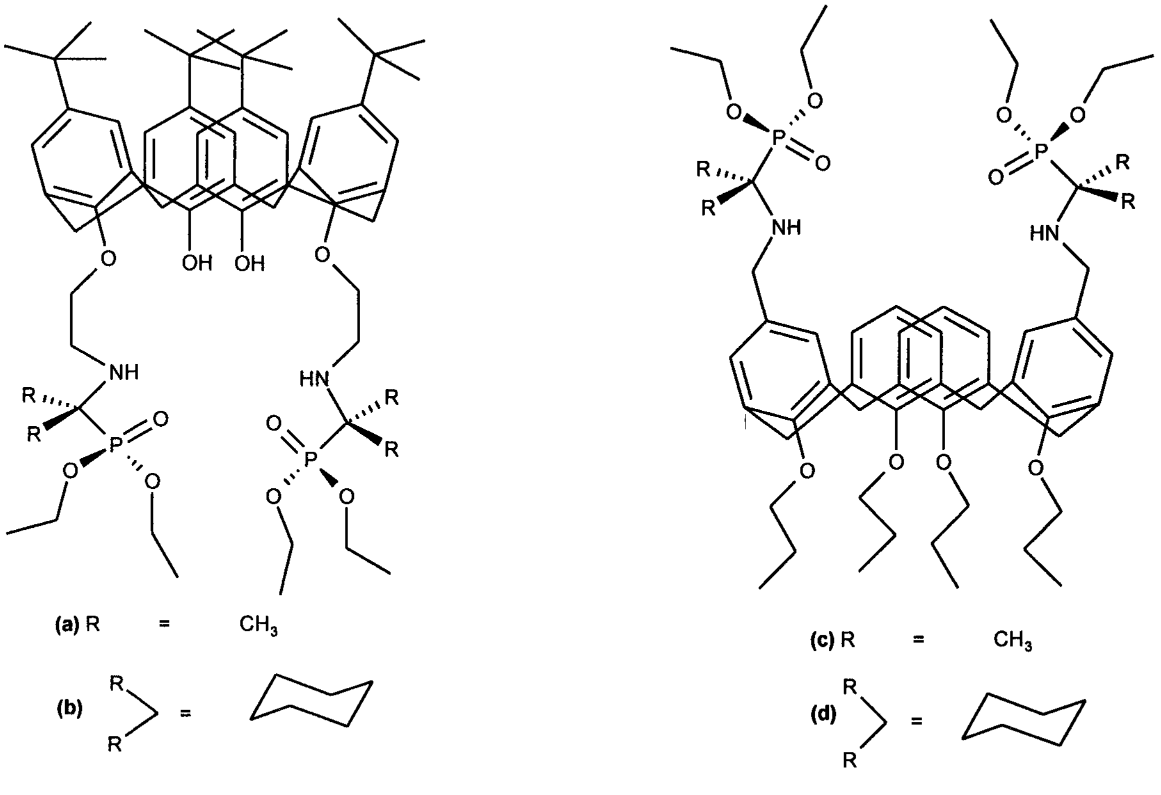

**Fig. 29:** Calix[4]arene based $\alpha$-amino phosphonates

Unlike carrier (Fig. 29, **a**) the molecular cavity of upper rim substituted calixarene (Fig. 29,**c**) can participate in complexation and recognize the aromatic side chains of amino acids. As a result, selective transportation of some amino acids through membrane is enhanced. For example, carrier (Fig. 29, **c**) transports phenylalanine 40 times faster than tryptophan. This proves that

1) The molecular cavity of calixarene is involved in complexation
2) The three points interaction of amino acid (due to carboxylate, ammonium groups and side chain) with the carrier leads to the enhancing of transport efficiency and selectivity.

Nishimura *et al.* reported /77/ the synthesis of chiral calix[4]arene analog (Fig. 30) and their properties for the chiral extraction and transport of amino acids. Glycine, L-alanine, and α- alanine were not extracted at all. On the other hand, L-phenyl alanine and L-tryptophan were effectively extracted with all receptors. Moreover, L-phenylalanine apparently extracted more than L-tryptophan because the indole ring was too large to fit the cavity formed by the pendant groups of the receptors. The order of this extraction efficiency was also explained by the hydrophobicity of amino acids Phe>Trp>Ala>Gly. The selectivity order of transport for each amino acid is nearly equal to that of the extraction. L-Amino acids, which strongly interact with receptors, can be enantioselectively transported more than D-ones. Receptor which had phenylglycine group possessing the phenyl group directly to the chiral center showed the highest transport efficiency and enantioselectivity among all receptors in order to clearly recognize the structure of guest molecule.

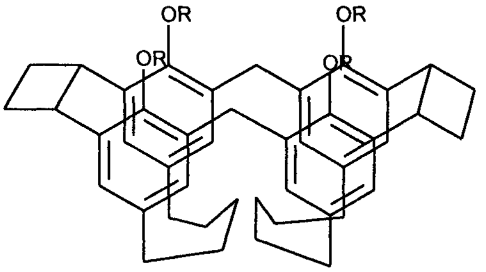

**(a)** R = $CH_2CONHCHCOOMe$ (with $CH_2Ph$ on the CH)

**(b)** = $CH_2CONHCHPhCOOMe$

**(c)** = $CH_2COOCHPh$ (with $CH_3$ on the CH)

**Fig. 30:** Calix[4]arene derived esters

During the last decade, the synthesis of azamacrocyclic compounds has received considerable attention because of their relationship to biomimetic and catalytic systems and the applications of this type of chelating agents to biology and medicine. Complexes of macrocyclic or polydentate ligands with pendant arms were reviewed and represented as models for biological molecules. A critical survey of molecular recognition of DNA, RNA and related biomolecules by complexes with aza-macrocyclic ligands has also been done /78/. The chiral calix(aza)crowns comprising a calixarene and a chiral polyamine derived from an amino acid were synthesized and their chiral recognition abilities and applications of host (Fig. 31, **a**) as anion receptors were explored /79/. The dibenzoate or racemic tartaric acid and racemic amygdalic acid were chosen as probes. $^1H$ NMR spectra indicated that the interaction of host with the L- and D- forms of the tartaric acid derivatives are different, resulting in two singlet resonances (at δ 6.03 and 5.99, J = 12 Hz) for CH protons of racemic tartaric acid.

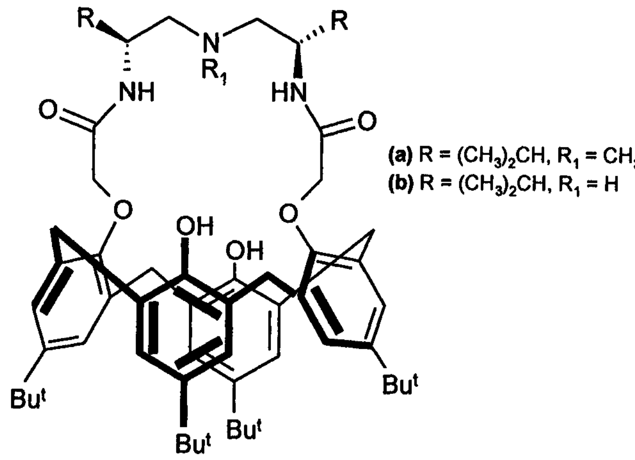

**Fig. 31:** Calix[4](aza)crown

The interaction between the host (Fig. 31, **a**) and the racemic tartaric acid led to a partial cone conformation of the host in the complex, because an appropriate environment is beneficial to the complexation of the relatively larger molecular skeleton of tartaric acid in comparison with racemic amygdlic acid. This conclusion was based on the appearance of two signals at δ 37.2 and 31.8 ppm in the $^{13}C$ NMR spectrum of the complex. A series of UV- absorption experiments was also undertaken. The association constant K for the D- and L-form guests was calculated as $9.83 \times 10^3$ $M^{-1}$ and $5.04 \times 10^3$ $M^{-1}$, respectively. All these results indicated that interaction between the host and guest do occur, which could be due to the formation of H- bonds or π- π interaction between host and guest and host (Fig. 31, **a**), has the ability to recognize the D- and L- forms of a tartaric acid derivative differently and has different recognition conformations with different guest species.

Enantioselective recognition of chiral antipodes is considered to be one of the most interesting and challenging tasks in modern chemistry, because of many applications in bio-sciences and implications for understanding life processes. Three novel bicyclo peptidocalix[4]arene derivatives were synthesized /79/ and used for chiral coatings for gas sensing to discriminate the enantiomers of methyl lactate. Evaluation of the ability of these calixarene derivatives to act as chiral sensor coatings discriminating between the enantiomers of methyl lactate was achieved using a QCM technique. At the same concentration, the (R)-enantiomer showed significantly larger sensor responses than the (S)-enantiomer. Paritition coefficient K values ($K = C_f/C_g$, where $C_g$ is gas concentration in the air and $C_f$ is that in the film) were found to be larger for (R)-enantiomers than for the (S)-enantiomers, revealed that this coatings were more sensitive for the (R)-enantiomers. Studies on various analytes can me made in this direction /80/.

Calix[4]arenes functionalized with urea on their upper wider rims have self-complementary recognition sites and assemble into dimeric form. The selectivity for heterodimerization is exclusive, with amino acids that have β branched side chains such as iso-leucine and valine. This exclusive recognition is capable of discriminating between enantiomers of guest molecules /81/. Ballistreri *et al.* synthesized ureidocalix[5]arene and it was used as receptors for multipoint molecular recognition of aminoacids and biogenic amines /82/.

Cyclic peptides provide useful tools for the study of protein secondary structure and of the structure-activity relationships of bioactive peptides. They also play a significant role in ion transport across biological membranes /83/, in self-assembling size adjustable nanotubes /84/ and in serving as enzyme models /85/ or as artificial receptors /86,87/. They have also reduced conformational flexibility compared to their parent linear molecules, but still possess quite large conformational flexibility. Several constraints such as non-natural amino acids /88/, S-S linkages /89,90/ and semi-rigid aromatic units /89-91/ have been incorporated in cyclic backbone. Thus, the first synthesis of the lower rim linked calix[4]arene bearing cyclic peptides was reported by Cheng *et al.* /92/ for reducing not only conformational freedom but also providing additional binding sites. Binding experiments were performed with disodium 4-nitrophenyl phosphate in order to test the potential of macrocyclic host in molecular recognition. For comparison, binding constants with three reference receptors, i.e. cyclopeptide and two disubstituted low rim calix[4]arenes were determined. Results showed calix[4]arene cyclopeptides as much more efficient receptors than the three reference receptors, demonstrating that the introduction of a calix[4]arene unit substantially enhances molecular recognition for a phosphate group, and the amide groups are mainly responsible for the recognition. Thus, the properties of cyclic peptides are improved.

Because of the accessibility and biological relevance, amino acids and peptide moieties were incorporated into calixarenes in order to achieve chirally modified macrocyclic ligands. Only very few multicyclic receptor systems (mentioned above) combining with cyclopeptide and calix[4]arene have been synthesized /61a/, characterized and presented as good candidates in future studies of chiral recognition and chiral catalysis /93/.

Hydrogen bonding is of great importance for the stabilization of the shapes of large biological molecules, such as cellulose, proteins and nucleic acids /94/. In biological systems the co-operative action of peptidic hydrogen bonds plays a major role in organization, assembly and molecular recognition processes. Even self-assembly is a powerful tool for the understanding, modelling and mimicking of biological systems. Synthetic self assembled models were reviewed with biological functions /95/.

With the supramolecular approach to a synthetic combination of rigid molecular scaffolds that act as topological templates with amino acids or small peptides as organizational elements, various constrained systems such as cavitands /96/, steroids /97/, porphyrins /98/, constrained peptides /99/ and sugars /100/ have been used. Surprisingly, calix[4]arenes /101/ have been used only infrequently as topological templates in combination with amino acids or peptides /102/. The synthesis of calix[4]arene hybrid receptors functionalized at the upper rim with L-alanine and dipeptide units has been reported /56a/. Very little work has been done on the upper /61a/ or lower rim /102/ calix[4]arenes tetra-(o-amino acid or dipeptide) derivatives that describe their organization by intramolecular hydrogen bonds. X-ray analysis of two biomimetic complexes revealed that hydrogen bonding and CH/π interactions between the guest ligand I and the calix[6]arene host structure play a key role in their stabilization /56/.

Zinic *et al.* presented /103/ hydrogen bonding motifs of calix[4]arene derivatives (Fig. 32) with one, two or four stands at the lower rim and with one amino acid unit each. Intra- and interstrand hydrogen bonding was observed for structurally related cavitands with strands at the lower rim incorporate peptidic functionalities.

Rebek *et al.* reported the synthesis and association properties of a series of ureido calix[4]arenes N-linked to α- amino acids /81,104/. In these examples, urea-urea self-assembly takes place exclusively with isoleucine and valine, other amino acids giving rise to association only with simple

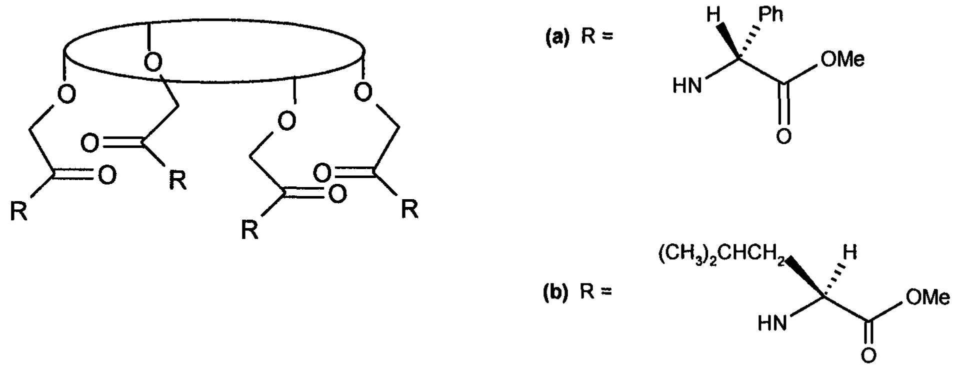

**Fig. 32:** Tetra substituted calix[4]arene amino acid derivatives phenylureidocalix[4]arenes(heterodimers). The effect of extended peptide chains on the dimerization of ureidocalix[4]arenes has never been investigated before. So Mendoza *et al.* /105/ demonstrated the dimerization of calix[4]arene ureidopeptides for the first time. The synthesis and association behavior of calix[4]arenes having four leucines (octylamide at the C- terminus) was linked to the urea groups at the upper rim and having four dipeptide $^{L}$Leu-$^{D}$Leu-OMe chain on the top of the ureas.

Although the dimerization phenomenon was not observed in polar solvent, the addition of only a few percent of polar solvent would result in complete disassembly to monomers /81,106/. It was desirable to prepare systems that might associate in polar solvent, especially for the development of compounds that would be compatible with a biological environment and have applications in drug transport and delivery, biocatalysis, and the binding and detection of biomolecules. So, the first calix[4]arene derivative was reported by Brewster *et al.* /107/ that undergoes hydrogen-bond-mediated self-association in polar, protic solvent. The association constant $K_a$, for this dimerization was found to be 29000 $M^{-1}$ in 24:1 MeOH:$H_2O$, which was higher than the $K_a$ value that was reported for a peptidylureidocalixarene in apolar solvent. This tetrapeptidocalix[4]arene contained amino acids attached to the upper rim through the carboxyl termini. The dimerization and recognition properties of these new class of peptidocalixarenes were studied by NMR and mass spectroscopy. It was found that the addition of arginine and lysine to the calixarene solution results in the formation of 1:1 inclusion complexes of the amino acid with the calixarene and dissociation of the calixarene dimer, rather than the encapsulation of the amino acid within the dimer. The addition of the guest molecule resulting in the disassembly of calixarene dimers was not reported previously. This study may lead to the use of calixarenes for molecular recognition in biologically relevant environments.

Nomura *et al.* /109/ synthesized calixarenes carrying amino acids at the lower rim. They studied the effect on the binding ability of intramolecular hydrogen bonding in the hydrophilic pseudocavities of these calixarenes towards metal ions such as $Na^+$ and $Ag^+$. The results revealed that the introduction of amino acids such as glycine, L-alanine, L-valine and L-phenyl alanine gives stabilization to the binding sites of calixarenes. Nomura and his coworkers /109/ extended their studies on the polymorphism of p-tert-butyl calix[4]arene derivatives having amino acid derivatives at the lower rim to stabilize their hydrophilic pseudo cavity by circular intramolecular hydrogen bonding. Sone *et al.* /110/ prepared chiral bishomodiaza calix[4]arenes containing amino acid residues. Circular dichroism spectra showed that intramolecular

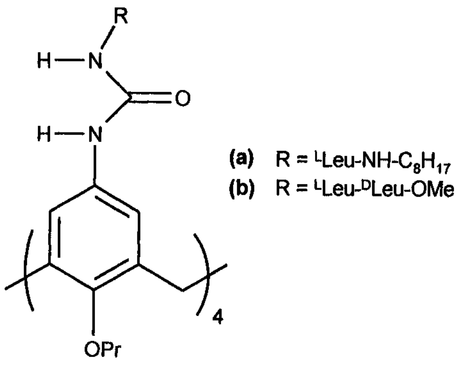

**Fig. 33:** Calix[4]arene ureidopeptides hydrogen bonding plays an important role in the transmission of the chirality from the amino acid residues to the cyclophane unit.

A high degree of functionality and conformational flexibility of calixarenes makes the isolation and characterization of products a difficult task. However, the conformational freedom affords the possibility of formation of enzyme models for mono copper sites complexes. It was shown that the rigidified preorganized cavity structure of calix[6]arenes is imperative for guest recognition. By introduction of amino acid ester groups onto the platform of calix[6]arene, non covalent interaction may probably contribute to the conformational stability of calix[6]arene derivatives. In addition, chiral centers may provide an asymmetric environment for their guests. By keeping this in view, Huang *et al.* achieved chiral modification of the skeleton of 1,3,5-trimethoxy-p-tert-butyl calix[6]arene by amino acid ester residues. They also studied their conformational behaviour and concluded that the conformations are greatly influenced by the amino acid ester residues /111/.

### 5. CONCLUDING REMARKS

Calixarenes are versatile molecules and can behave as potential enzyme mimics. In the first part of this article, functional enzyme models that mimic di- and tri- nuclear metallo phosphodiesterases have been reviewed. However, these functional models were not proved to be efficient compared to natural enzymes but they focus more light on to possible catalysis mechanism employed by these natural analogues. These types of studies have provided a base or platform for the future design of a cleaving agent for RNA or other related type of molecules. Mimics for enzymes such as transferase, ATPase, aldolase or heme mimics were also presented and the extent of their potency as functional models was discussed.

In the subsequent part, a few chemical systems containing Cu(I), Cu(II) or Zn(II) ions were presented as models for metal active sites in enzymes. These synthetic models with a suitable design are able to provide new insights into the structures, spectroscopic properties and activities of metalloprotein active site.

The last part of the review contains the discussion of molecular recognition of biorelevant species such as amino acids and their transport studies using various calixarene derivatives, especially those with amino acid linkages at either rim. The role and importance of hydrogen bonding to stabilize biomolecules can also be understood by amino acid substituted calixarenes.

**ACKNOWLEDGEMENTS**

One of the authors (Y. K. Agrawal) is thankful for the financial assistance given by the Chemical Society, London; DST, New Delhi; GMDC Science and Research Centre, Ahmedabad.

**REFERENCES**

1. F. M. Menger, *Proc. Natl. Acad. Sci. (USA)*, **99**, 4818 (2002).
2. N. J. Turro, *J. Chem. Soc., Chem. Commun.*, **2002**, 2279.
3. C. D. Gutsche, *Acc. Chem. Res.*, **16**, 161 (1983).
4. R. Breslow, S. D. Dong, *Chem. Rev.*, **98**, 1997 (1998).
5. V. Stastny, P. Lhotak, V. Michlova, I. Stibor, J. Sykora, *Tetrahedron*, **58**, 7207 (2002).
6. A. Casnati, C. Massera, N. Pelizzi, I. Stibor, E. Pinkhassik, F. Ugozzoli, R. Ungaro, *Tetrahedron Lett.*, **43**, 7311 (2002).
7. P. Molenveld, S. Kapsabelis, J. F. J. Engbersen, D. N. Reinhoudt, *J. Am. Chem. Soc.*, **119**, 2948 (1997).
8. P. Molenveld, W. M. G. Stikvoort, H. Kooijman, A. L. Spek, J. F. J. Engbersen, D. N. Reinhoudt, *J. Org. Chem.*, **64**, 3896 (1999).
9. P. Molenveld, J. F. J. Engbersen, H. Kooijman, A. L. Spek, D. N. Reinhoudt, *J. Am. Chem. Soc.*, **120**, 6726 (1998).
10. P. Molenveld, J. F. J. Engbersen, D. N. Reinhoudt, *J. Org. Chem.*, **64**, 6337 (1999).
11. P. Molenveld, J. F. J. Engbersen, D. N. Reinhoudt, *J. Org. Chem.*, **64**, 3269 (1999).
12. P. Molenveld, J. F. J. Engbersen, D. N. Reinhoudt, *Angew. Chem. Int. Ed. Engl.*, **38**, 3189 (1999).
13. (a) M. Yashiro, A. Ishikubo, M. Komiyama, J. *Chem. Soc., Chem. Commun.*, **1997**, 83.
    (b) C. Bazzicalupi, A. Bencini, A. Bianchi, V. Fusi, C. Giorgi, P. Paoletti, B. Valtancoli, D. Zanchi, *Inorg. Chem.*, **36**, 2784 (1997).
14. S. Liu, A. D. Hamilton, *Bioorg. Med. Chem. Lett.*, **7**, 1779 (1997).
15. (a) N. H. Williams, J. Chin, *J. Chem. Soc., Chem. Commun.*, **131** (1996).
    (b) S. Seog, N. D. Sung, R. S. Hynes, J. Chin, *Inorg. Chem.*, **35**, 7472, (1996).
16. K. G. Ragunathan, H. J. Schneider, *Angew. Chem. Int. Ed. Engl.*, **35**, 1219 (1996).
17. (a) R. Hettich, H. J. Schneider, *J. Chem. Soc., Perkin Trans. 2*, 2069 (1997).
    (b) R. Hettich, H. J. Schneider, *J. Am. Chem. Soc.*, **119**, 5638 (1997).
    (c) M. Komiyama, J. Sumaoka, K. Yonezawa, Y. Matsumoto, M. Yashiro, *J. Chem. Soc., Perkin Trans. 2*, **1997**, 75.
    (d) J. Rawlings, A. C. Hengge, W. W. Cleland, *J. Am. Chem. Soc.*, **119**, 75, (1997).
18. (a) B. F. Baker, H. Khalili, N. Wei, J. R. Morrow, *J. Am. Chem. Soc.*, **119**, 8749 (1997).
    (b) K. Bracken, R. A. Moss, K. G. Ragunathan, *J. Am. Chem. Soc.*, **119**, 9323 (1997).
    (c) J. Sumaoka, A. Kajimura, M. Ohno, M. Komiyama, *Chem. Lett.*, 507 (1997).
    (d) T. C. Bruice, A. Tsubouchi, R. O. Dempcy, L. P. Olson, *J. Am. Chem. Soc.*, **118**, 9867 (1996).

19. P. Molenveld, J. F. J. Engbersen, D. N. Reinhoudt, *Chem. Soc. Rev.*, **29**, 75 (2000).
20. D. M. Perreault, E. V. Anslyn, *Angew. Chem. Int. Ed. Engl.*, **36**, 433 (1997).
21. M. Komiyama, K. Yoshinari, *J. Org. Chem.*, **62**, 2155 (1997).
22. G. M. Bonora, S. Drioli, F. Felluga, F. Mancin, P. Rossi, P. Scrimin, P. Tecilla, *Tetrahedron. Lett.*, **44**, 535, (2003).
23. S. Liu, A. D. Hamilton, *Tetrahedron Lett.*, **38**, 1107 (1997).
24. B. Linkletter, J. Chin, *Angew. Chem. Int. Ed. Engl.*, **34**, 472 (1995).
25. E. L. Hegg, K. A. Deal, L. L. Kiessling, J. N. Burstyn, *Inorg. Chem.*, **36**, 1715 (1997).
26. T. Itoh, H. Hisada, T. Sumiya, M. Hosono, Y. Usui, Y. Fujii, *J. Chem. Soc., Chem. Commun.*, **1997**, 677.
27. M. J. Young, J. Chin, *J. Am. Chem. Soc.*, **117**, 10577 (1993).
28. J. Yan, R. Breslow, *Tetrahedron Lett.*, **41**, 2059 (2000).
29. (a) E. I. Solomon, P. Chen, M. Metz, S. K. Lee, A. E. Palmer, *Angew. Chem. Int. Ed. Engl.*, **40**, 4570 (2001).
    (b) A. Messerschmidt, *Struct. Bonding*, **90**, 37 (1998).
30. (a) K. Brown, M. Tegon, M. Prudencio, A. S. Pereira, S. Besson, J. J. Moura, I. Moura, C. Cambillau, *Nat. Struct. Biol.*, **7**, 191 (2000).
    (b) M. L. Alvarez, J. Ai, W. Zumft, J. Sanders-Loehr, D. M. Dooley, *J. Am. Chem. Soc.*, **123**, 576 (2001).
31. D. J. E. Spencer, Bryan J. Johnson, Brian. J. Johnson, W. B. Tolman, *Organic Letters*, **4**, 1391 (2002).
32. G. Dospil, J. Schatz, *Tetrahedron Lett.*, **42**, 7837 (2001).
33. T. Gunnlaugsson; R. J. H, Davies; M. Nieuwenhuyzen; C. S. Stevenson; R. Viguier; S. Mulready, *J. Chem. Soc., Chem. Commun.*, **2002**, 2136.
34. W. C. Putnam, J. K. Bashkin, *J. Chem. Soc., Chem.Commun.*, **2000**, 767.
35. M. Komiyama, K. Isaka, S. Shinkai, *Chemistry Letters*, **1991**, 937.
36. Y. T. Ming, Y. Z. Feng, W. Li, G. J. Ying, Y. S. De, S. X. Fa, *Spectrochimica Acta Part A*, **58**, 3033 (2002).
37. G. Ashkenasy, D. Margulies, C. E. Felder, A. Shanzer, L. S. Powers, *Chem. Eur. J.*, **8**, 4017 (2002).
38. D. J. Cram, K. D. Stewart, I. Goldberg, K. N. Trueblood, *J. Am. Chem. Soc.*, **107**, 2574 (1985).
39. C. D. Gutsche, M. Iqbal, K. S. Nam, K. See, I. Alam, *Pure & Applied Chem.*, **60**, 483 (1988).
40. Y. Molard, C. Bureau, H. P. Lopez, R. Lamartine, J. B. R. de Vains, *Tetrahedron Lett.*, **40**, 6383 (1999).
41. Y. Molard, H. P.- Lopez, *Tetrahedron Lett.*, **42**, 4799 (2001).
42. W. Kaim, J. Rall, *Angew. Chem. Int. Ed. Engl.*, **35**, 43 (1996).
43. L. L. Clainche, M. Giorgi, O. Reinaud, *Eur. J. Inorg. Chem.*, 1931 (2000).
44. J. P. Klinman, *Chem. Rev.*, **96**, 2541 (1996).
45. S. Blanchard, L. L. Clainche, M. N. Rager, B. Chansou, J. P. Tuchagues, A. F. Duprat, Y. L. Mest, O. Reinaud, *Angew. Chem. Int. Ed. Engl.*, **37**, 2732 (1998).
46. Y. Rondelez, M. N. Rager, A. Duprat, O. Reinaud, *J. Am. Chem. Soc.*, **124**, 1334 (2002).

47. Y. Rondelez, O. Seneque, M. N. Rager, A. F. Duprat, O. Reinaud, *Chem. Eur. J.*, **6**, 4218 (2000).
48. (a) J. L. Atwood, D. L. Clark, R. K. Juneja, G. W. Orr, K. D. Robinson, R. L. Vincent, *J. Am. Chem. Soc.*, **114**, 755 (1992).
    (b) R. Castro, L. A. Godinez, C. M. Criss, S. M. Bott, A. E. Kaifer, *J. Chem. Soc., Chem. Commun.*, **1997**, 935.
    (c) R. Castro, L. A. Godinez, C. M. Criss, A. E. Kaifer, *J. Org. Chem.*, **62**, 4928 (1997).
    (d) J. Alvarez, Y. Wang, M. Gomez-Kaifer, A. E. Kaifer, *J. Chem. Soc., Chem. Commun.*, **1008**, 1455.
49. Y. Rondelez, G. Bertho, O. Reinaud, *Angew. Chem. Int. Ed. Engl.*, **41**, 1044 (2002).
50. L. L. Clainche, M. Giorgi, O. Reinaud, *Inorg. Chem.*, **39**, 3436 (2000).
51. (a) W. N. Lipscomb, N. Strater, *Chem. Rev.*, **96**, 2375 (1996).
    (b) D. S. Auld, *Metal Sites in Proteins and Models In Structure and Bonding*, Ed. P. J. Sadler, Springer- Verlag: Berlin- Heidelberg, **89**, p. 29 (1997).
52. G. Parkin., *J. Chem. Soc., Chem. Commun.*, **20**, 1971 (2000).
53. E. Kimura, T. Koike, M. Shionoya, *Metal Sites in Proteins and Models In Structure and Bonding*, Ed. P. J. Sadler, Springer- Verlag: Berlin- Heidelberg, **89**, p. 1-28 (1997).
54. (a) M. Ruf, F. A. Schell, R. Walz, H. Vahrenkamp, *Chem. Ber.*, Recl., **130**, 101 (1997).
    (b) C. Bergquist, G. Parkin, *Inorg. Chem.*, **38**, 422 (1999).
55. O. Seneque, M. N. Rager, M. Giorgi, O. Reinaud, *J. Am. Chem. Soc.*, **122**, 6183 (2000).
56. O. Seneque, M. Giorgi, O. Reinaud, *J. Chem. Soc., Chem. Commun.*, **2001**, 984.
57. O. Seneque, Y. Rondelez, L. L. Clainche, C. Inisan, M. N. Rager, M. Giorgi, O. Reinaud, *Eur. J. Inorg. Chem.*, 2597 (2001).
58. A. P. Davis, R. S. Wareham, *Angew. Chem. Int. Ed. Engl.*, **38**, 2978 (1999).
59. H. J. Schneider, E. Eblinger, M. Sirish, *Adv. Supramol. Chem.*, **6**, 185 (2000).
60. J. Budka, M. Tkadlecova, P. Lhotak, I. Stibor, *Tetrahedron*, **56**, 1883 (2000).
61. (a) F. Sansone, S. Barboso, A. Casnati, M. Fabbi, A. Pochini, F. Ugozzoli, R. Ungaro, *Eur. J. Org. Chem.*, 897 (1998).
    (b) F. Sansone, S. Barboso, A. Casnati, D. Sciotto, R. Ungaro, *Tetrahedron Lett.*, **40**, 4741 (1999).
62. (a) J. Scheerder, M. Fochi, J. F. J. Engbersen, D. N. Reinhoudt, *J. Org. Chem.*, **59**, 7815 (1994).
    (b) J. Scheerder, J. F. J. Engbersen, A. Casnati, R. Ungaro, D.N. Reinhoudt, *J. Org. Chem.*, **60**, 6448 (1995).
63. G. Arena, A. Contino, F. G. Gulino, A. Magri, F. Sansone, D. Sciotto, R. Ungaro, *Tetrahedron Lett.*, **40**, 1597 (1999).
64. G. Arena, A. Casnati, L. Mirone, D. Sciotto, R. Ungaro, *Tetrahedron Lett.*, 38, 1999 (1997).
65. G. Arena, A. Casnati, A. Contino, D. Sciotto, R. Ungaro, *Tetrahedron Lett.*, **38**, 4685 (1997).
66. G. Arena, A. Contino, F. G. Gulino, A. Magri, D. Sciotto, R. Ungaro, *Tetrahedron Lett.*, **41**, 9327 (2000).
67. C. S. Wilcox, *Frontiers in Supramolecular Chemistry and Photochemistry*, H. J. Schneider, H. Durr, Eds., VCH, Weinheim, 123 (1991).

68. A. Arduini, M. Fabbi, M. Mantovani, L. Mirone, A. Pochini, A. Secchi, R. Ungaro, *J. Org. Chem.*, **60**, 1454 (1995).
69. M. Lazzarotto, F. Sansone, L. Baldini, A. Casnati, P. Cozzini, R. Ungaro, *Eur. J. Org. Chem.*, **2001**, 595.
70. A. Casnati, M. Fabbi, N. Pellizzi, A. Pochini, F. Sansone, R. Ungaro, *Biorg. Med. Chem. Lett.*, **6**, 2699, 1996.
71. L. Frish, F. Sansone, A. Casnati, R. Ungaro, Y. Cohen, *J. Org. Chem.*, **65**, 5026 (2000).
72. F. Sansone, L. Baldini, A. Casnati, M. Lazzarotto, F. Ugozzoli, R. Ungaro, *Proc. Natl. Acad. Sci., USA*, **99**, 4842 (2002).
73. (a) K. Hiratani, T. Yamaguchi, *Liquid Membrane: Chemical Applications*, T. Araki, H. Tsukube Eds. CRC Press, Inc.: Florida, 103 (1990).
(b) C. Seel, A. Galan, J. de Mendoza, *Topics in Current Chemistry*, Ed. Weber, E., **175**, 102 (1995).
74. J. L. Sessler, A. Andrievsky, *J. Chem. Soc., Chem. Commun.*, **1996**, 1119.
75. I. S. Antipin, I. I. Stoikov, A. R. Garifzyanov, A. I. Konovalov, *Zh. Obsch. Khim.*, **66**, 402 (1996).
76. I. S. Antipin, I. I. Stoikov, E. M. Pinkhassik, N. A. Fitseva, I. Stibor, A. I. Konovalov, *Tetrahedron Lett.*, **38**, 5865 (1997).
77. Y. Okada, Y. Kasai, J. Nishimura, *Tetrahedron Lett.*, **36**, 555 (1995).
78. J. Costamagna, G. Ferraudi, B. Matsuhiro, M. C. Vallette, J. Canales, M. Villagran, J. Vargas, M. J. Aguirre, *Coord. Chem. Rev.*, **196**, 125 (2000).
79. Y. He, Y. Xiao, L. Meng, Z. Zeng, X. Wu, C. T. Wu, *Tetrahedron Lett.*, **43**, 6249 (2002).
80. W. Guo, J. Wang, C. Wang, J. Q. He, X. He, J. P. Cheng, *Tetrahedron Lett.*, **43**, 5665 (2002).
81. R. K. Castellano, C. Nuckolls, J. Rebek Jr., *J. Am. Chem. Soc.*, **121**, 11156 (1999).
82. F. P. Ballistreri, A. Notti, *Org. Lett.*, **5**, 1071 (2003).
83. T. J. Marrone, K. M. Merz Jr., *J. Am. Chem. Soc.*, **114**, 7542 (1992).
84. T. D. Clark, L. K. Buehler, M. R. Ghadiri, *J. Am. Chem. Soc.*, **120**, 615 (1998).
85. H. Ishida, K. Donowaki, M. Suga, K. Shimose, K. Ohkubo, *Tetrahedron Lett.*, **36**, 8987 (1995).
86. D. Ranganathan, V. Haridas, K. P. Madhusudanan, R. Roy, R. Nagaraj, G. B. John, M. B. Sukhaswami, *Angew. Chem. Int. Ed. Engl.*, **35**, 1105 (1995).
87. (a) W. C. Still, *Acc. Chem. Res.*, **29**, 155 (1996).
(b) P. D. Bailey, D. G. W. Clarke, G. A. Crofts, *J. Chem. Soc., Chem. Commun.*, **1992**, 658.
88. M. D. Struthers, R. P. Cheng, B. Imperial, *Science*, **271**, 342 (1996).
89. (a) L. J. Mu, J. P. Cheng, H. Huang, J. M. Lu, X. B. Hu, *Synth. Commun.*, **28**, 3029 (1998).
(b) H. Huang, L. J. Mu, J. P. Cheng, J. M. Lu, X. B. Hu, *Synth. Commun.*, **28**, 4036 (1998).
90. D. Ranganathan, V. Haridas, I. L. Karle, *J. Am. Chem. Soc.*, **120**, 2695 (1998).
91. R. Haubner, W. Schmitt, G. Holzemann, S. L. Goodman, A. Jonczyk, H. Kessler, *J. Am. Chem. Soc.*, **118**, 7881 (1996).
92. X. Hu, A. S. C. Chan, X. Han, J. He, J. P. Cheng, *Tetrahedron Lett.*, **40**, 7115 (1999).
93. X. Hu, J. He, A. S. C. Chan, J. P. Cheng, *Tetrahedron Asymmetry*, **10**, 2685 (1999).
94. L. Stryer, *Biochemistry*, 3rd ed.; W. H. Freeman and Co.: New York (1988).

95. R. Fiammengo, M. C. Calana, D. N. Reinhoudt, *Current Opinion in Chemical Biology*, **5**, 660 (2001).

96. C. B. Gibbs, A. R. Mezo, A. S. Canston, J. R. Fraser, F. C. S. Tsai, J. C. Sherman, *Tetrahedron*, **32**, 8719 (1995).

97. R. Hirschmann, P. A. Sprengler, T. Kawasaki, J. W. Leahy, W. C. Shakespeare, A. B. Smith III, *Tetrahedron*, **49**, 3665 (1993).

98. K. S. Akerfeldt, R. M. Kim, D. Camac, J. T. Groves, J. D. Lear, J. W. F. DeGrado, *J. Am. Chem. Soc.*, **114**, 9656 (1992).

99. P. Dumy, I. M. Eggleston, S. Cervigni, U. Sila, X. Sun, M. Mutter, *Tetrahedron Lett.*, **36**, 1255 (1995).

100. R. Hirshmann, K. C. Nicolau, S. Pietranico, E. M. Leahy, J. Salvino, B. Arison, M. Cichy, P. G. Spoors, W. C. Shakespeare, P. A. Sprengler, P. Hamely, A. B. Smith III, T. Beisine, K. Raynor, L. Maechler, C. Donaldson, W. Vale, R. M. Freidinger, R. Cascieri, C. D. Strader, *J. Am. Chem. Soc.*, **115**, 12550 (1993).

101. (a) C. D. Gutsche, *Aldrichimica Acta*, **28**, 3 (1995).
(b) V. Bohmer, *Angew. Chem. Int. Ed. Engl.*, **34**, 713 (1995).

102. M. S. Pena, Y. Zhang, S. Thibodeaux, M. L. McLaughlin, A. M. de la Pena, I. M. Warner, *Tetrahedron Lett.*, **37**, 5841 (1996).

103. L. Frkanec, A. Visnjevac, B. K. Prodic, M. Zinic, *Chem. Eur. J.*, **6**, 442, (2000).

104. R. K. Castellano, B. H. Kim, J. Rebek Jr., *J. Am. Chem. Soc.*, **119**, 12671, (1997).

105. A. M. Rincon, P. Prados, J. de Mendoza, *J. Am. Chem. Soc.*, **123**, 3493 (2001).

106. (a) Y. L. Cho, D. M. Rudkevich, A. Shivanyuk, K. Rissanen J. Rebek, Jr., *Chem. Eur. J.*, **6**, 3788 (2000).
(b) C. A. Schalley, R. K. Castellano, M. S. Brody,D. M. Rudkevich, G. Siuzdak, J. Rebek, Jr., *J. Am. Chem. Soc.*, **121**, 4568 (1999).
(c) O. Mogck, M. Pons, V. Bohmer, W. Vogt, *J. Am. Chem. Soc.*, **119**, 5706 (1997).

107. R. E. Brewster, S. B. Shuker, *J. Am. Chem. Soc.*, **124**, 7902 (2002).

108. E. Nomura, M. Takagaki, C. Nakaoka, M. Uchida, H. Taniguchi, *J. Org. Chem.*, **64**, 3151 (1999).

109. E. Nomura, M. Takagaki, C. Nakaoka, M. Uchida, H. Taniguchi, *J. Org. Chem.*, **65**, 5932 (2000).

110. K. Ito, T. Ohta, Y. Ohba, T. Sone, *J. Hetero. Chem.*, **37**, 79 (2000).

111. H. S. Yuan, Y. Zhang, Y. J. Hou, X. Y. Zhang, X. Z. Yang, Z. T. Huang, *Tetrahedron*, **56**, 9611 (2000).

